# Investigations on Regulation of MicroRNAs in Rice Reveal [Ca^2+^]_cyt_ Signal Transduction Regulated MicroRNAs

**DOI:** 10.3389/fpls.2021.720009

**Published:** 2021-10-18

**Authors:** Shivani Kansal, Vaishali Panwar, Roseeta Devi Mutum, Saurabh Raghuvanshi

**Affiliations:** Department of Plant Molecular Biology, University of Delhi South Campus, New Delhi, India

**Keywords:** calcium signaling, miRNAs, CAMTA TFs, dehydration, miR156a, miR167h

## Abstract

MicroRNAs (miRNAs) are critical components of the multidimensional regulatory networks in eukaryotic systems. Given their diverse spectrum of function, it is apparent that the transcription, processing, and activity of the miRNAs themselves, is very dynamically regulated. One of the most important and universally implicated signaling molecules is [Ca^2+^]_cyt_. It is known to regulate a plethora of developmental and metabolic processes in both plants and animals; however, its impact on the regulation of miRNA expression is relatively less explored. The current study employed a combination of internal and external calcium channel inhibitors to establishing that [Ca^2+^]_cyt_ signatures actively regulate miRNA expression in rice. Involvement of [Ca^2+^]_cyt_ in the regulation of miRNA expression was further confirmed by treatment with calcimycin, the calcium ionophore. Modulation of the cytosolic calcium levels was also found to regulate the drought-responsive expression as well as ABA-mediated response of miRNA genes in rice seedlings. The study further establishes the role of calmodulins and Calmodulin-binding Transcription Activators (CAMTAs) as important components of the signal transduction schema that regulates miRNA expression. Yeast one-hybrid assay established that OsCAMTA4 & 6 are involved in the transcriptional regulation of miR156a and miR167h. Thus, the study was able to establish that [Ca^2+^]_cyt_ is actively involved in regulating the expression of miRNA genes both under control and stress conditions.

## Introduction

Plants being sessile in nature need to face all the environmental and biotic challenges in one place. Plants modulate their internal system to cope with these challenges. Changes in the growth or environment of the plants are conveyed to the cell *via* primary messengers such as hormones while it is relayed to the nucleus *via* the secondary messengers such as cAMP, cGMP, Inositol triphosphate, diacylglycerol, and the most widely studied [Ca^2+^]_cyt_. Apart from these, players like hydrogen sulfide, hydrogen peroxide, carbon monoxide, and nitric oxide, collectively termed as “gasotransmitters,” have also emerged as potential signaling messengers in cells (Gaupels et al., 2011; Mittler et al., 2011; Lisjak et al., 2013). Their signaling cascade is often routed *via* [Ca^2+^]_cyt_ or MAPK signal transduction components. [Ca^2+^]_cyt_ as a second messenger of internal and external signals has been well-studied and documented in a plethora of studies and reviews, describing its role in signaling in various plant physiological functions such as abiotic stress response (Dodd et al., 2010), stomatal aperture (McAinsh et al., 1990), self-incompatibility (Franklin-Tong et al., 1993), pathogenic and symbiotic interactions (Ma and Berkowitz, 2007), and growth of pollen tube and root tips (Hepler et al., 2001). Upon perceiving an environmental stimulus or developmental cue, rapid and precise [Ca^2+^] oscillations are generated within the cytoplasm through the influx of [Ca^2+^] from internal and extracellular stores. Besides cytosolic [Ca^2+^] signatures and signaling, the plant cell nucleus has also been proved to contain within an independent Ca^2+^-signaling system (Pauly et al., 2000, 2001; Xiong et al., 2004) to regulate gene transcription. Calmodulin (CaM) is said to be the primary decoder of the signal along with the support of CaM-binding proteins in the nucleus. For instance, nuclear apyrase in pea binds to CaM and is activated by Ca^2+^/CaM complex (Hsieh et al., 2000); nuclear localized potato CaM-binding protein (PCBP) identified from developing potato tubers binds to CaM in a Ca^2+^-dependent manner (Reddy et al., 2002). The strongest evidence for the theory is the identification of nuclear transcription factors that are regulated directly or indirectly by CaM (Yang and Poovaiah, 2003). For instance, CAM7 in *Arabidopsis* has been demonstrated to regulate photomorphogenic growth and light-responsive gene expression by binding to Z-/G-box elements in their promoters, including CAB1 and RBCS1A. CAM7 was also shown to work independently yet in an interdependent pathway with HY5 to regulate the photomorphogenic growth of *Arabidopsis* (Kushwaha et al., 2008). WRKY class of TFs has also been shown to interact with Ca^2+^/CaM complex, especially the WRKY group IId (Park et al., 2005). Another separate class of transcription factors known as CAMTAs (Calmodulin-binding transcription activators), described to be evolutionarily conserved from humans to plants (Finkler et al., 2006), responds rapidly to multiple abiotic stresses such as drought, salinity, heat, cold, and UV, thus regulating the signaling required (Yang and Poovaiah, 2003). Despite the name, CAMTAs can act as transcriptional activators as well as repressors; as CAMTA3 was demonstrated for activating the expression of *CBF2*-imparting cold tolerance (Doherty et al., 2009), as well as repressing the expression of *EDS1*, a regulator of the salicylic acid level (Du et al., 2009). Recently, AtCAMTA3 has been shown to repress the genes involved in SA-biosynthesis and SA-mediated immunity in healthy plants (Kim et al., 2017).

In biological systems, microRNAs (miRNAs) represent a significant and critical layer of regulation of protein-coding genes at the post-transcriptional level. In our previous studies and others as well, we have seen that several miRNAs can express differentially to a similar cue in different rice cultivars, thus emphasizing the evolution of distinct regulatory mechanisms controlling miRNA expression in different cultivars (Mutum et al., 2013; Kansal et al., 2015; Balyan et al., 2017). Such unique regulation would have a cascading effect on plant biology since a single miRNA regulates several protein-coding genes. Thus, the study of the regulation of miRNA expression is of critical importance. MiRNAs have been shown to be differentially regulated by different stages of growth and development, biotic and abiotic stresses, etc. At the molecular level, miRNAs have been shown to respond to secondary messengers of signaling in various publications. A study showed seven miRNAs, including miR169, miR397, miR1425, miR408-5p, and miR827 that were upregulated, while miR319a-3p and miR408-5p were downregulated by H_2_O_2_ stress (Li et al., 2011). Similarly, miRNAs were also shown responsive to exogenously supplied H_2_S, and it was established that their expression under drought simulation was mediated *via* H_2_S (Shen et al., 2013). Analysis in the *lcd* (knockdown mutant of H_2_S-producing enzyme L-CYSTEINE DESULFHYDRASE) mutants under control and drought-simulated conditions showed lower expression levels of some miRNAs like miR167a/c/d, miR393a, miR396a, miR398a/b/c, which could be rescued by resupplying H_2_S. This confirmed that H_2_S regulates miRNA activity to modulate the drought response of *Arabidopsis*. Recently, miRNAs have been shown to be differentially regulated during early embryo development under conditions of calcium deficiency in the growth medium in peanuts (Chen et al., 2019). The targets of such miRNAs included seed/embryo development-related genes, cell-division and proliferation-related genes, and phytohormone signaling-related genes. However, to the best of our knowledge, the demonstration that miRNAs are responsive to [Ca^2+^]_cyt_ has not been made till now.

In the current study, we attempt to establish a stronger link between calcium signaling and its control over the regulation of miRNAs in rice. The miRNAs were confirmed to be calcium responsive using miRNA expression tools [small RNA libraries and quantitative real-time PCR (qRT-PCR)] under conditions of calcium scarcity (created using Ca^2+^ channel inhibitors) and excess of [Ca^2+^]_cyt_ using a Ca^2+^ ionophore A23187. Furthermore, the involvement of Ca^2+^ signaling in differential expressions of miRNAs under abiotic stress such as dehydration and hormonal stress such as ABA was also shown. Additionally, we show here the participation of calmodulins and CAMTAs in the regulation of miRNA expression. Furthermore, our results show physical interaction between OsCAMTA4 and OsCAMTA6 and the promoters of miR156a and miR167h, thereby strengthening the evidence of hold of calcium signaling over miRNA regulation.

## Methods

### Rice Plant Growth and Stress Treatment

Rice seeds [*O. sativa* subsp. *indica* var Nagina22 (N22)] were obtained from the Indian Agricultural Research Institute (IARI) Pusa, New Delhi. Seeds were sterilized and planted on a muslin cloth tied to a tray filled with a rice growth medium (as described in Kansal et al., 2015). These were grown for 1 week in a culture room with 28°C ± 2°C temperature and 16-h/8-h day/night settings and then subjected to various stresses. For seedling stress experiments, the seedlings were transferred to a solution of calcium channel inhibitors [mix of LaCl_3_ (Lanthanum chloride, 5 mM), Verapamil (50 μM), LiCl (Lithium chloride, 5 mM), Ruthenium Red (100 μM) for 3 h], calcium ionophore A23187 (10 μM for 5 h), TriFluoPerazine (200 μM for 6 h) or ABA (Abscisic acid, 100 μM for 3 h). All these solutions and control (MQ water) contained the surfactant Silwett at 0.01% final concentration for better and uniform absorption. All the chemicals were obtained from Sigma Aldrich.

For dehydration stress, the seedlings were taken out of the respective solution, dabbed with tissue paper to remove excess liquid sticking to the roots, and kept open in the air for air-drying until leaf rolling was observed. Whole seedlings were frozen in liquid nitrogen and stored at −80°C.

Drought stress at the mature stage was given as per Mutum et al. (2016). Briefly, drought stress was given by withdrawing water supply to the field bed 10 days before the “mean heading date” of the cultivar. The samples were collected from plants that had attained heading, displayed leaf rolling, and had soil moisture content dropped below 15% (as measured using Hydra Probe Soil Moisture Sensor). Tissue (flag leaf and inflorescence) was frozen in liquid nitrogen *in situ* and stored at −80°C. Induction of drought was checked by estimating transcript levels of drought stress marker genes such as Rubisco small subunit *(RBCS)* and *OsbZIP23* in the collected tissue as described in Mutum et al. (2016).

### RNA Isolation and cDNA Synthesis

RNA was isolated using TriReagent (Sigma) as per the protocol of the manufacturer. It was subjected to DNaseI treatment followed by small RNA enrichment by the 4M LiCl method as given in Kansal et al. (2015). For cDNA synthesis, 2 μg of RNA was incubated with 100 pmoles of either oligodT primer (Eurofins Genomics) for total RNA or miR_oligodT primer (Eurofins Genomics) for small RNA using the SuperscriptII cDNA synthesis kit (Invitrogen). All the primers have been enlisted in Supplementary Table 1.

### Small RNA Library Preparation, Quality Check, and Analysis

Small RNA libraries were prepared with 2 μg of enriched small RNA using NEBNext® Small RNA Library Prep Set for Illumina® (Multiplex Compatible) (New England Biolabs) according to the protocol of the manufacturer in biological duplicates. Each biological replicate consisted of a set of 5–6 seedlings individually subjected to each step of stress, RNA isolation, library preparation, and sequencing. Briefly describing the library preparation, 3′ and 5′ small RNA adapters were ligated to the small RNA population, sequentially followed by First Strand cDNA synthesis. After this, the Small RNA primer for Illumina along with index primers was added, and the sample was subjected to PCR. The cDNA prepared was thus run on 6% PAGE gel for size excision of miRNA band (~140 nts) and purified further. The libraries prepared were thus checked for quality using 2100 Bioanalyzer High Sensitivity DNA chip (Agilent) according to the protocol of the manufacturer. The libraries were sequenced using the Illumina platform by Sandor LifeSciences Pvt Ltd. Small RNA Library Analysis was done using CLC Genomics Workbench version 7.0.4 (Qiagen) with the help of the “Small RNA Analysis” tool. For a further detailed analysis of differentially expressed genes, the “Statistical Analysis” tool, in particular, “Empirical Analysis of DGE” which uses “Edge” as the tool for calculating differentially expressed genes, was used. The statistically significant DEGs were filtered at having *p* ≤ 0.05 and expression fold change of 2X in stress condition vs. the control.

### Quantitative Real-Time PCR

The first-strand cDNA synthesized (section RNA isolation and cDNA synthesis) was used for qRT-PCR with appropriate dilution. The reaction was set up by adding TaqMan Master Mix chemistry (2x, Applied Biosystems). The reaction was run in the ABI StepOnePlus Real-Time system as per its default cycling for TaqMan chemistry. Rice actin and 5S rRNA were used as endogenous controls for protein-coding gene and miRNA profiling, respectively. For each sample, at least three technical and three biological replicates were set up. The expression fold change was calculated as stress vs. control with 5S rRNA used as the internal control for normalization purposes. The use of ^*^ above a bar in the bar graphs depicts its significance as ^*^p ≤ 0.05 as recorded by the *t*-test. A two-tailed *t*-test (with the assumption of unequal variance) has been used to determine if there is a significant difference between the means of control and treatment groups.

### Mirna-Target Analysis and Classification

Putative targets of miRNAs were selected from in-house analyzed publicly available rice degradome libraries (GSM455939, GSM455938, GSM434596, GSM960648, and GSM476257; (Wu L. et al., 2009; Li et al., 2010; Zhou M. et al., 2010; Wang et al., 2012) and in-house generated three additional degradome libraries from spikelets at the heading and anthesis stage, flag leaf at the heading stage of N22 (Mutum et al., 2016). The targets falling within the criteria of category 0–2 along with read no ≥10 and *p* ≤ 0.05 were selected as putative targets. Category “0” is defined as >1 raw read at the position, with abundance at a position equal to the maximum on the transcript, and with only one maximum on the transcript. Category “1” is described as >1 raw read at the position, with abundance at the position equal to the maximum on the transcript, and more than one maximum position on the transcript. Category “2” includes >1 raw read at the position, and abundance at the position less than the maximum but higher than the median for the transcript (Yang et al., 2013).

The target loci were then used to extract their GO annotations from the RGAP database and classified according to their GO term of “molecular function.” Similarly, the loci were checked for their presence in the RiceCyc database available at the Gramene database.

The promoter sequences (2 kb upstream of the precursor start site) were retrieved from IRDb (Indica Rice Database, www.genomeindia.du.ac.in/irdb) and subjected to a sequence-based motif search for the calcium-responsive motifs enlisted in literature (Galon et al., 2010).

### Yeast One-Hybrid Assay

Yeast one-hybrid assay was performed using the EZ yeast transformation kit (MP Biomedicals) as per the instructions. For the Y1H promoter construct, 110 and 200 bp regions were selected from 2-kb promoter regions of MIR156 and MIR167, respectively, that contained at least one CAMTA-binding site (Supplementary Figure 1). These mentioned promoter fragments were PCR amplified from the genomic DNA of N22 and cloned into the pAbAI vector as bait, harboring the aureobasidin (AUR1-C) reporter gene. Full-length CDS of *OsCAMTA4* and *OsCAMTA6* were PCR amplified from genomic DNA of N22 and cloned into the pGADT7-AD vector as prey. The miR156a-pAbAi, miR167h-pAbAi, OsCAMTA4-pGADT7, and OsCAMTA6-pGADT7 plasmids were linearized and co-transformed into the Y1H gold yeast strain. The transformants were selected on SD medium-lacking Uracil (U) and Leucine (L) (SD/-L-U), respectively, as per the protocol of the manufacturer. Colonies obtained were screened for a positive insert *via* colony PCR. Furthermore, the positive clones were inoculated as primary culture and subsequently used in the secondary culture (3 ml) and grown at 30°C, 200 rpm for 3 h till the OD = 1 at 600 nm. Each culture was then serially diluted (10^−1^, 10^−2^, and 10^−3^), and droplets of 10 μl of each dilution, including the undiluted culture, were placed on the selection media (SD/-L-U) and incubated at 30°C for 3–5 days till the formation of colonies. The interaction was determined based on the ability of co-transformed yeast cells to grow on dropout media-lacking Uracil and Leucine with 100, 200, and 300 ng/ml of Aureobasidin A.

### Accessions

Accession IDs for camta mutants used in the study are as follows: SALK_108806C (AtCAMTA1), SALK_064889C (ATCAMTA3), SALK_087870C (ATCAMTA4), SALK_120516C (ATCAMTA5), and SALK_078900C (ATCAMTA6). The small RNA libraries are accessible at NCBI under accession PRJEB47136 and IBDC (Indian Biological Data Centre) accession INRP000017.

## Results

### miRNAs Are Responsive to [Ca^2+^]_cyt_ in N22 Rice

**Figure 1:**
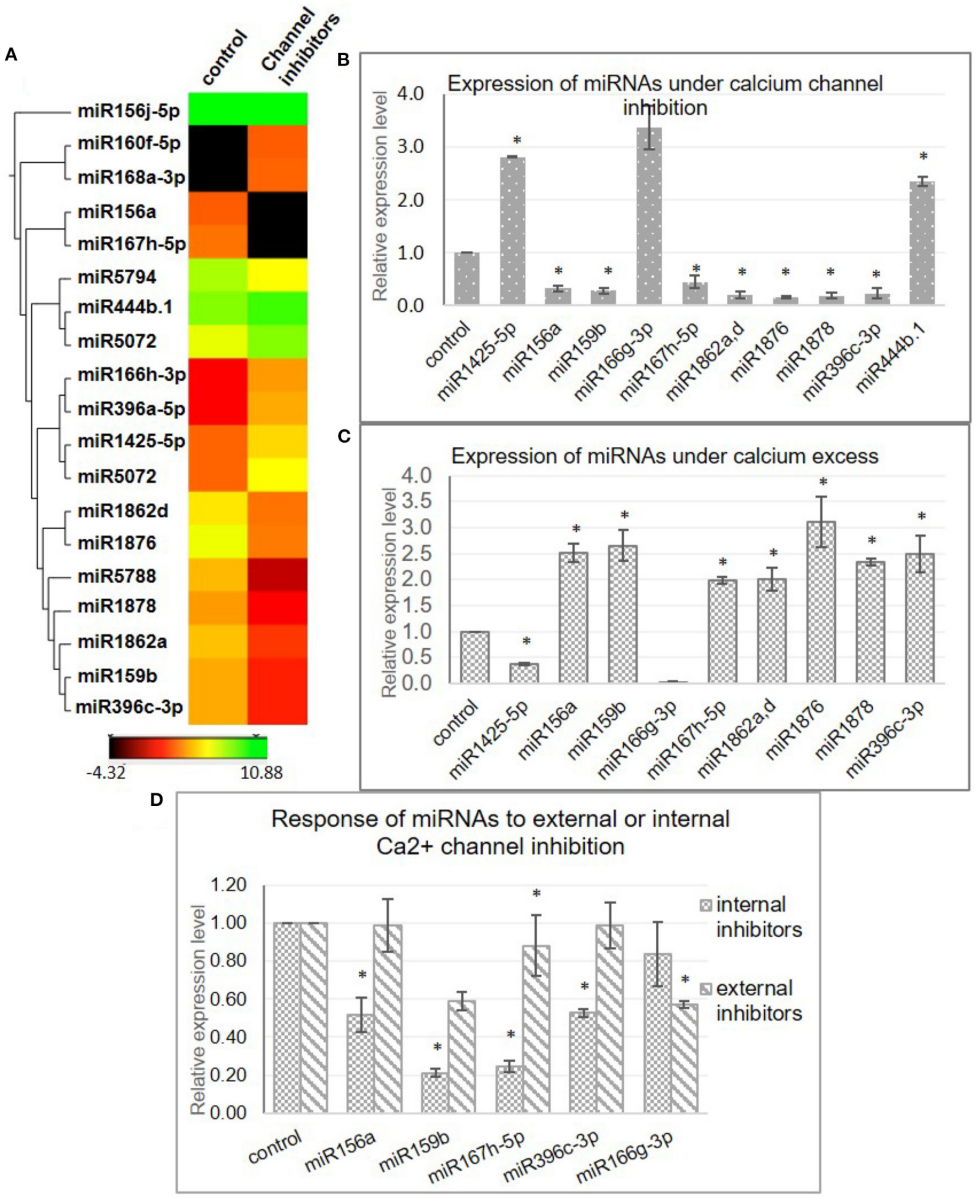
Identification of [Ca^2+^]_cyt_-responsive microRNA (miRNAs) in N22. **(A)** Hierarchical clustering of miRNAs detected with significant fold change in the presence of [Ca^2+^]_cyt_ channel inhibitors. The heatmaps show the expression values of miRNAs as detected in the NGS data in the control and inhibitor-treated samples. **(B)** Validation of [Ca^2+^]_cyt_ responsiveness of selected miRNAs by quantitative real-time PCR (qRT-PCR) analysis. **(C)** Confirmation of [Ca^2+^]_cyt_ responsiveness by qRT-PCR-generated expression profiles under calcium ionophore treatment. **(D)** qRT-PCR-generated expression profiles of select miRNAs under treatment with a specific set of Ca^2+^ channel blockers that block either the intracellular Ca^2+^ channels (RR and LiCl) or extracellular Ca^2+^ channels (Verapamil and LaCl_3_). Asterisks denote significant change as observed by the *t*-test.

Cellular calcium levels are primarily regulated by the activity of the calcium channels present either on the plasma membrane (blocked by LaCl_3_ and Verapamil) and/or the mitochondrial (inhibited by Ruthenium Red) or endoplasmic reticulum membrane (blocked by LiCl). These are well-characterized inhibitors and have been previously experimentally shown to alter the cellular calcium levels in plants (Knight et al., 1996; Sedbrook et al., 1996; Legué et al., 1997; Hu et al., 2004; Song et al., 2011; Gao and Zhang, 2019; Zheng et al., 2020). In order to identify miRNA genes that are regulated by [Ca^2+^]_cyt_, a cocktail of Ca^2+^ channel blockers (viz. LaCl_3_, LiCl, Ruthenium Red, and Verapamil) was used to treat young rice seedlings, followed by sequencing of the sRNA population. The efficiency of the inhibitor treatment was confirmed by monitoring the expression of a calcium-responsive “Ca^2+^-dependent protein kinase” OsCPK6 (Wan et al., 2007) (Supplementary Figure 2). Briefly describing, 293 and 303 miRNAs were detected in control libraries, while 258 and 279 miRNAs were detected from the tissue treated with calcium channel inhibitors (Supplementary Table 2). Furthermore, comparative analysis of the datasets revealed 17 differentially expressed miRNAs (fold change ≥ 2 and *p* ≤ 0.05) (Supplementary Table 3); out of which, 10 were found to be downregulated, while 7 were upregulated in the presence of Ca^2+^ channel inhibitors (Figure 1A). Subsequently, qRT-PCR analysis reaffirmed the calcium inhibitor-dependent differential expression of 11 miRNAs in rice (miR1425-5p, miR156a, miR159b, miR166g-3p, miR167h-5p, miR1862a,d, miR1876, miR1878, miR396c-3p, and miR444b.1) (Figure 1B).

The [Ca^2+^]_cyt_ inducibility of these miRNAs was further verified with the help of a Ca^2+^ ionophore, i.e., A23187 which is expected to reverse the expression pattern obtained with inhibitors. A23187, also known as calcimycin, is an ionophore that binds to Ca^2+^ ions and acts as their carrier by inserting itself in the plasma membrane, thereby acting as a channel for the divalent. Quantitative real-time PCR profiling under control and ionophore treatment confirmed nine miRNAs (miR1425-5p, miR156a, miR159b, miR167h-5p, miR1862a,d, miR1876, miR1878, and miR396c-3p) that responded to channel inhibitors as well as to the ionophore but in inverse fashion, thereby confirming their [Ca^2+^]_cyt_ inducibility (Figure 1C).

To identify the source of [Ca^2+^]_cyt_ inducibility of these miRNAs, subsequent analysis was done by treating rice seedlings with only internal or extracellular channel inhibitors to decipher the independent involvement of internal and external calcium stores in this regulation (Figure 1D). Interestingly, miR156a, miR159b, miR167h-5p, and miR396c-3p were significantly downregulated when internal channels were inhibited but showed no remarkable change with the blocking of plasma membrane channels. On the other hand, miR166g-3p was downregulated in the presence of external inhibitors only. Thus, it goes to show that specific miRNAs are uniquely regulated by the internal or external calcium reserves indicative of the complexity of the regulatory phenomenon.

However, from these data, it appears that the effect of [Ca^2+^]_cyt_ is not global since only a small proportion of miRNAs was found to be responsive, indicative of a transcriptional route (rather than miRNA processing where a higher proportion of miRNAs would have been differentially changed). Thus, dissecting closely, the [Ca^2+^]_cyt_ responsiveness of the three main miRNA-processing enzymes, namely DCL1, HYL1, and SE, was checked, and the response was found neither significant nor confirmed by ionophore treatment (Supplementary Figure 3).

Besides, we also found various calcium-responsive *cis*-elements in the promoter regions of miRNAs. The promoter sequences of some miRNAs were subjected to a search for the calcium-responsive motifs enlisted in literature (Galon et al., 2010). These motifs are ABA-related ABRE elements [(T/C) *ACGTG* (T/G)], CAMTA-binding sites [(C/A) *CGCG* (T/G/C) and (C/A) *CGTG*T], [Ca^2+^]_cyt_-responsive elements [CARE- (C/A) *ACGTG* (T/G/C) and (C/A) *ACGCG* (T/G/C)], E-box [C*ANNTG]*, G-box [C*ACGTG]*, Z-box [AT*ACGTG*T], GT-box [GGTAATT], and Rapid Stress Responsive Elements [*CGCG*TT]. For instance, MIR1425 has two ABRE, three CAMTA, and five E-box elements; MIR156A has one CARE and two CAMTA sites, and MIR159B has nine E-box sites.

### Targets of [Ca^2+^]_cyt_-Regulated miRNAs in N22 Are Functionally Widespread

To analyze the impact of these [Ca^2+^]_cyt_ regulated miRNAs, data from eight degradome libraries, including both in-house generated and publicly available, were analyzed to identify miRNA targets (Mutum et al., 2016). Targets identified under the category “0-2” and ≥10 degradome reads were considered for the analysis (Table 1).

**Table 1:** Targets of [Ca^2+^]_cyt_-responsive miRNAs in N22 as per degradome data.

**miRNA**	**Target loci**	**Target description**	**Read no**	**Catagory**	***P*-value**
miR1425-5p	LOC_Os10g35240	Rf1, mitochondrial precursor	280	0	0.0050
	LOC_Os10g35436		26	0	0.0054
	LOC_Os03g09110	Mitochondrial carrier protein	11	0	0.0845
miR156a	LOC_Os01g69830	OsSPL2—SBP-box gene family member	67	0	0.0608
	LOC_Os02g04680	OsSPL3—SBP-box gene family member	48	0	0.0132
	LOC_Os11g30370	OsSPL19—SBP-box gene family member	45	0	0.0608
miR159b	LOC_Os01g59660	MYB family transcription factor	172	0	0.0191
miR160f-5p	LOC_Os06g47150, LOC_Os10g33940	Auxin response factor 18	1067	0	0.0262
			1110	0	0.0262
	LOC_Os04g43910, LOC_Os02g41800	Auxin response factor	51	0	0.0262
			33	0	0.0262
miR166g-3p	LOC_Os03g01890, LOC_Os10g33960,	START domain containing protein	500	0	0.0242
			123	0	0.0242
miR167h-5p	LOC_Os02g06910	Auxin response factor 6	239	0	0.0277
	LOC_Os12g41950, LOC_Os06g46410	Auxin response factor	46	0	0.0312
			21	0	0.0312
	LOC_Os07g33790	Glutamate receptor 3.4 precursor	27	2	0.4066

*Targets of [Ca^2+^]_cyt_-responsive miRNAs in N22 were identified as per degradome data. Targets having read no ≥ 10 and cat between 0 and 2 were considered. Read number represents the number of reads identified at the predicted cut site in the degradome data, while the category represents the number of such predictive cut sites, having an abundance of reads mapping to the position (refer to Material and Method for details). P-value represents the probability of random mapping of the reads at the predictive cut site*.

**Figure 2:**
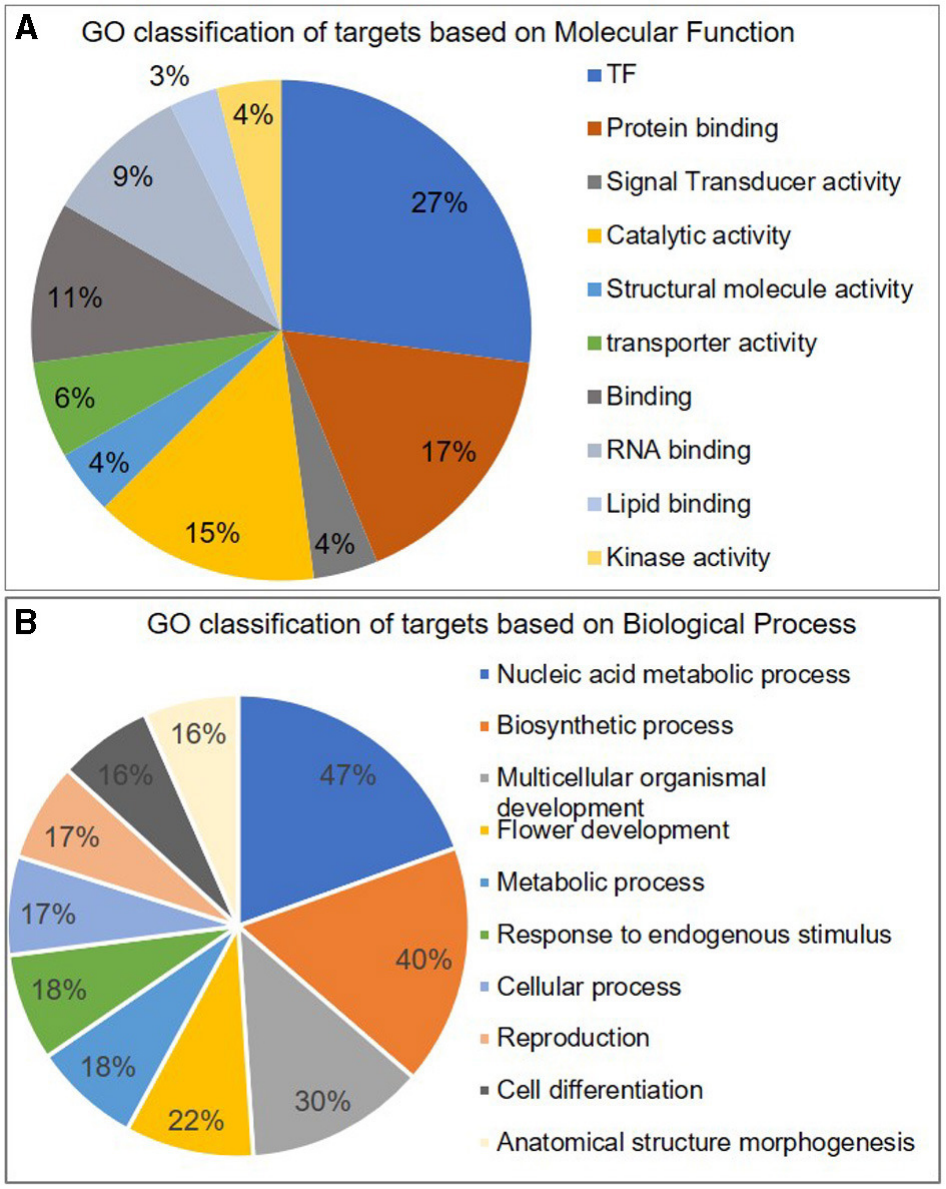
Classification of miRNA targets. Pie-chart representing different categories of miRNA targets classified according to their GO term of **A** molecular function and **B** biological process.

These targets included some well-known genes, such as certain transcription factors like *OsSPL* (miR156a), *MYB* [miR159b and START domain containing (miR166), *auxin response factors* (miR160, miR167). A gene ontology classification of these targets (based on molecular function) shows transcription factors being the major category along with protein-binding proteins and catalytic activity. The targets are widespread along almost all major categories like lipid and RNA-binding, kinase activity, transporters, structural molecule activity, etc. (Figure 2). Similarly, the majority of targets are related to the “biological process” terms like nucleic acid metabolism, biosynthetic process, multicellular organismal development, flower development, and metabolic processes.

### Dehydration and ABA Response of miRNAs Are Mediated by [Ca^2+^]_cyt_

**Figure 3:**
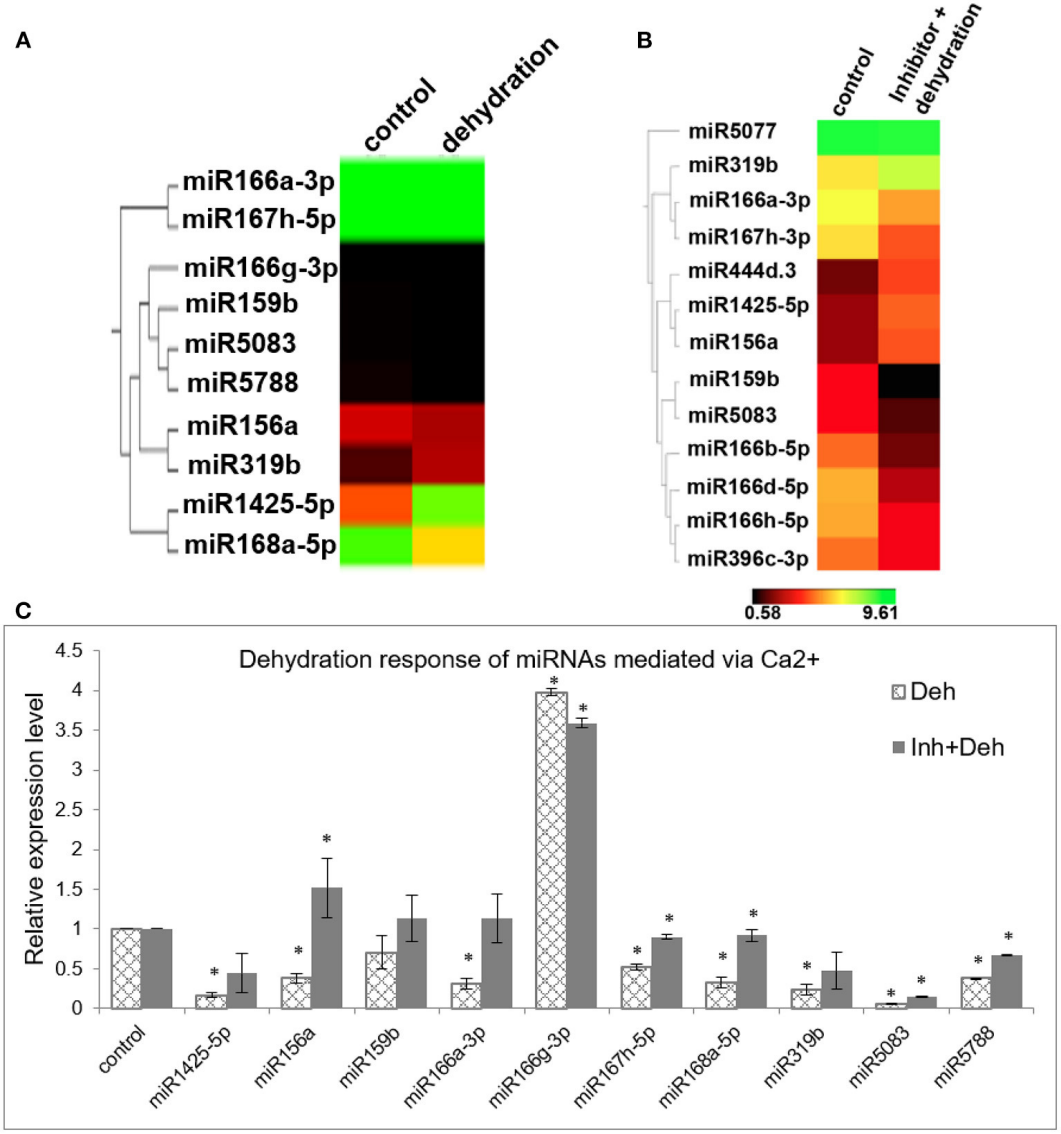
[Ca^2+^]_cyt_ mediates dehydration response of miRNAs in N22. **(A)** Hierarchical clustering of miRNAs with significant fold change between control and dehydrated seedlings; **(B)** control and dehydrated seedlings pretreated with [Ca^2+^]_cyt_ channel inhibitors; **(C)** confirmation of calcium-mediated dehydration response by qRT-PCR expression profiling of selected miRNAs. The asterisks denote significant change as observed by the *t*-test.

Since it is a well-known fact that miRNAs respond to various abiotic stresses and that [Ca^2+^]_cyt_ plays a significant role in relaying these abiotic stress cues, we investigated whether the dehydration response of miRNAs in rice is mediated by [Ca^2+^]_cyt_. Thus, 7-day-old rice seedlings were subjected to air dehydration in the presence or absence of the calcium channel inhibitor cocktail as described previously. Subsequently, sRNA libraries were generated and sequenced (Supplementary Tables 2, 4). Comparison of both the datasets gives us an insight into [Ca^2+^]_cyt_-mediated dehydration response of miRNAs. Consequently, through qRT-PCR confirmation, it was possible to identify that dehydration response of miR156a, miR167h-5p, miR168a-5p, miR5083, and miR5788 is being mediated by [Ca^2+^]_cyt_ levels (Figures 3A–C). Interestingly, miR156a and miR167h-5p also showed [Ca^2+^]_cyt_ responsiveness under control conditions.

**Figure 4:**
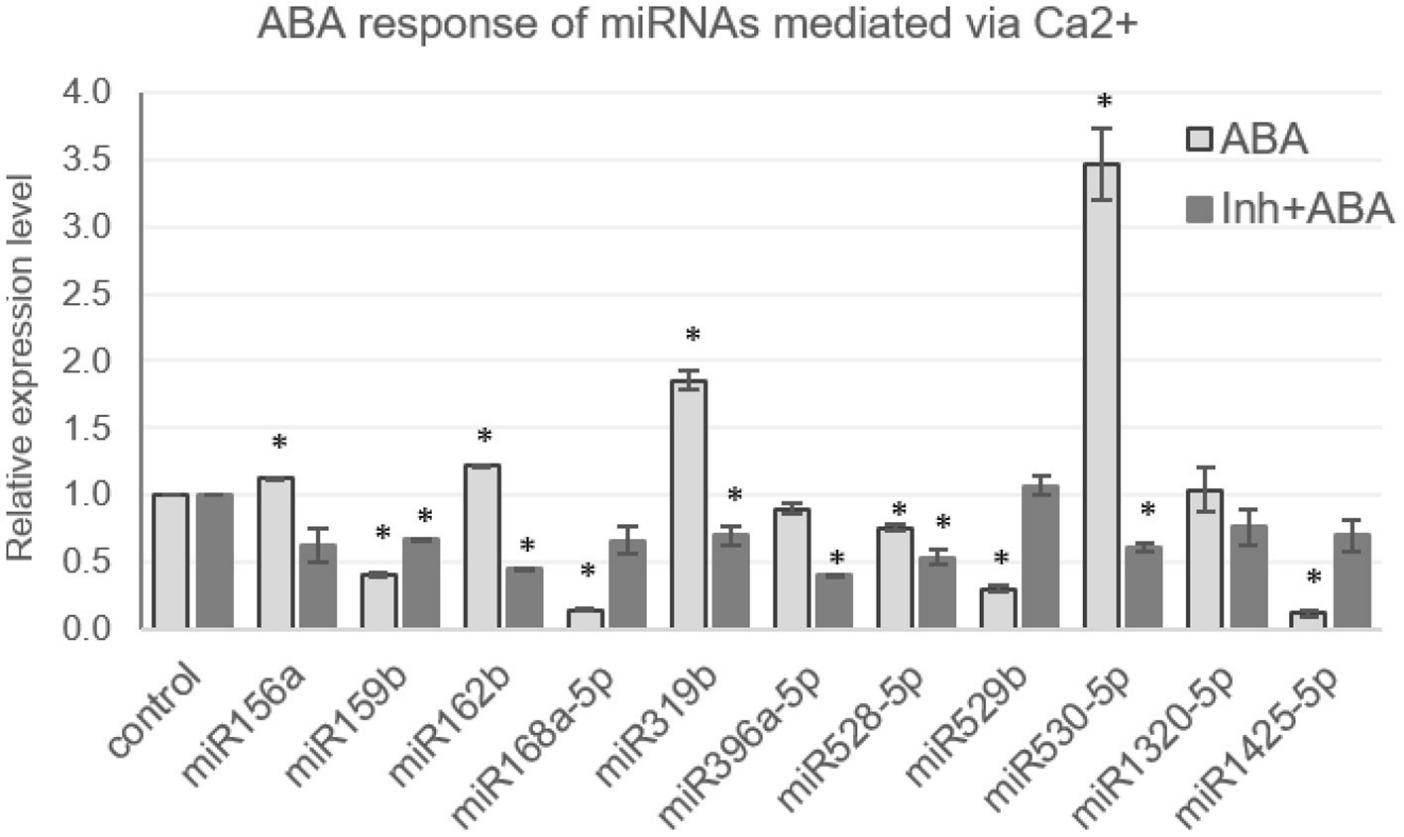
[Ca^2+^]_cyt_ mediates the ABA response of miRNAs in N22. The qRT expression profile of miRNAs in seedlings under control conditions, treated with ABA (100 μM) or treated with calcium channel inhibitors, followed by ABA (100 μM), has been shown. The asterisks denote significant change as observed by the *t*-test.

Another important signaling molecule involved in the regulation of gene expression is abscisic acid (ABA), aka the stress hormone that mediates several developmental processes in plants, including dormancy, abscission, seed germination, as well as the abiotic and biotic stress response. ABA levels increase during certain stress conditions and mediate adaptive response. Thus, experiments were conducted to assess the response of miRNAs to elevated ABA levels by treating 7-day-old seedlings with ABA (100 μM). Based on previous literature, a selected set of miRNAs was profiled, and the results indicated that miR1425-5p, miR159b, miR168a-5p, and miR529b are significantly downregulated, while miR319b and miR530-5p are upregulated in the presence of ABA (Figure 4).

Expression of miR156a, miR162b, miR1320-5p, miR396a, miR528-5p did not deviate much from control. The correlation of the ABA and dehydration responsiveness indicates that miR1425-5p and miR168a-5p are downregulated during dehydration and ABA. miR156a is downregulated by dehydration but is not ABA responsive. On the other hand, miR162b, miR396a-5p, miR528-5p, and miR1320-5p turn out neither dehydration nor ABA responsive. [Ca^2+^]_cyt_ was found to mediate the ABA response of miRNAs as well, since pre-blocking [Ca^2+^]_cyt_ channels alleviated the hormone-induced response of some miRNAs, such as miR159b, miR319b, and miR530-5p (Figure 4). Notably, miR159b is responsive to [Ca^2+^]_cyt_ in resting the state as well; however, its response to dehydration was not significant. Hence, here is a sample of demonstration of the versatile roles that [Ca^2+^]_cyt_ plays in regulating miRNAs under variable environmental conditions.

### Calmodulin and Calmodulin-Binding Transcriptional Activators (CAMTAs) Mediate the Expression of [Ca^2+^]_cyt_-Responsive miRNAs

**Figure 5:**
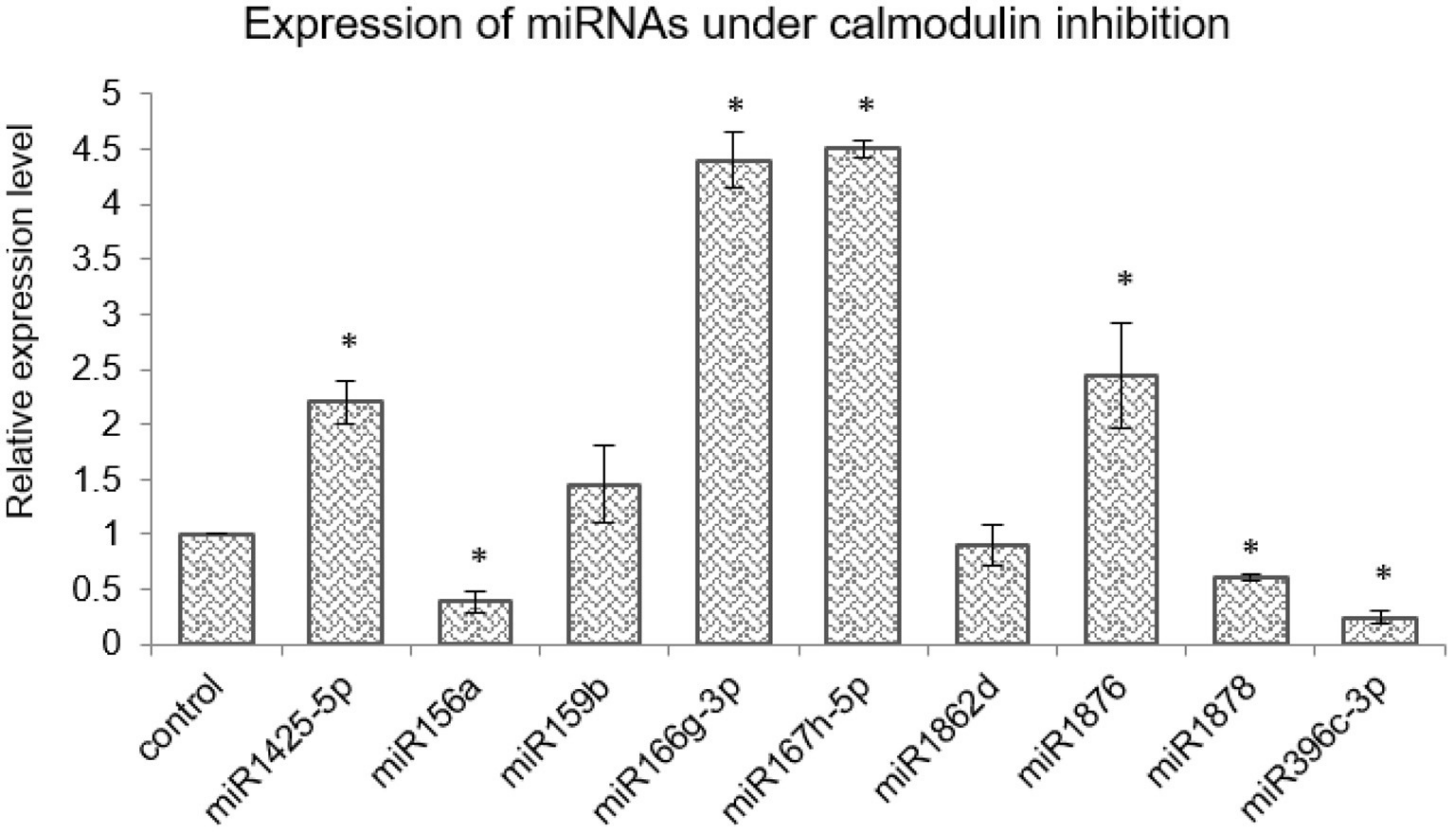
Calmodulins mediate miRNA expression in rice. qRT-PCR expression profiling of selected miRNAs in control and seedlings treated with the calmodulin inhibitor TFP (200 μM). The asterisk denotes significant change as observed by the *t*-test.

[Ca^2+^]_cyt_ cues are perceived by sensor-relay proteins such as calmodulins that, upon binding to [Ca^2+^]_cyt_ ions, change their conformation and relay the signal by binding to other proteins. Thus, as the next step, the expression of [Ca^2+^]_cyt_-responsive miRNAs were checked for their dependency on calmodulin. To achieve this, rice seedlings were treated with 200-μM calmodulin inhibitor Trifluoperazine (TFP). As per qRT-PCR profiles, three miRNAs namely, miR156a, miR1878, and miR396c-3p are downregulated, whereas miR1876, miR166g-3p, miR167h-5p, and miR1425-5p were upregulated upon calmodulin inhibitor treatment. Thus, CaM seems to positively relay the [Ca^2+^]_cyt_ signal to miR156a, miR1878, miR396c-3p, miR166g-3p, and miR1425-5p, while it appears to negatively affect miR1876 and miR167h-5p (Figure 5).

Subsequent to finding [Ca^2+^]_cyt_ and calmodulin-responsive miRNAs in rice, further components involved in mediating the [Ca^2+^] signal transduction of these miRNAs were explored. “Calmodulin-binding transcriptional activators” or CAMTAs are calcium-binding transcription factors that play an important role in mediating Ca^2+^/CaM-mediated gene expression. Five miRNAs viz. miR156a, miR160a-5p, miR166a-3p, miR167h-5p, and miR168a-5p are orthologous in both rice and *Arabidopsis* plus their promoter regions were found to have CAMTA-binding sites. To investigate whether CAMTAs regulate their expression, these miRNAs were profiled in the *Arabidopsis camta* mutants (available at the ABRC seed stock center). There are six CAMTA genes in *Arabidopsis*, and mutants specific to CAMTA loci *camta1, camta3, camta4, camta5*, and *camta6* were procured (Supplementary Figure 4; for accessions, refer to section Accessions).

**Figure 6:**
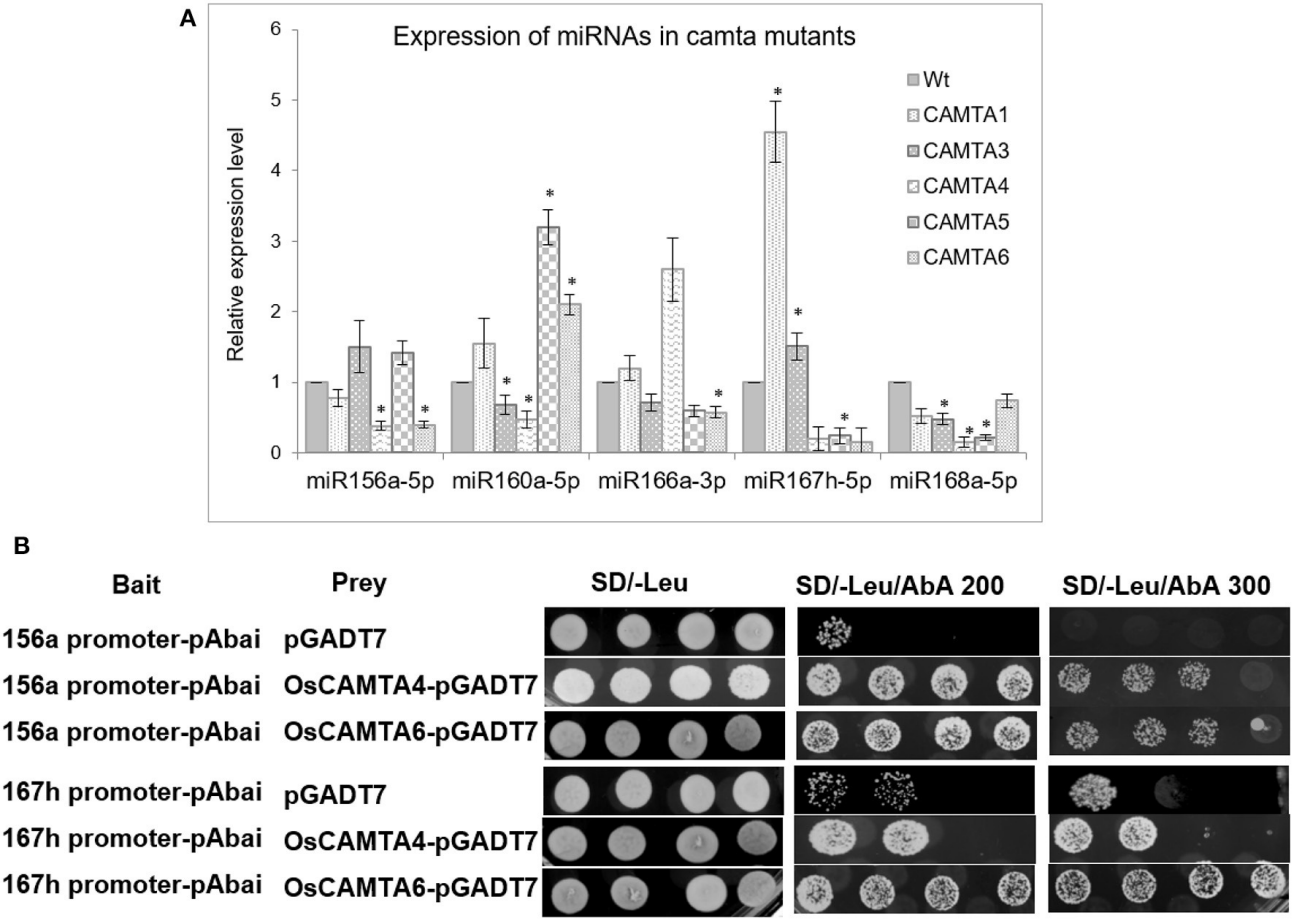
CAMTAs affect the expression of miRNAs. **(A)** The qRT-PCR profile of select miRNAs in wild type and the five *camta* mutants in *Arabidopsis*. **(B)** Yeast one-hybrid assay depicting the binding and trans-activation capability of OsCAMTA4 and OsCAMTA6 onto the promoters of *MIR156A* and *MIR167H*. The asterisks denote significant change as observed by the *t*-test.

Quantitative real-time profiling for these miRNAs in 10-day-old mutant seedlings revealed that miR156a is significantly reduced in *camta4* and *camta6* (Figure 6). Expression of miR160a-5p is reduced in *camta4* while enhanced in *camta5* and *camta6*. Similarly, miR168a-5p is reduced in *camta3,4,5* but not in *camta6*. On the other hand, miR167h-5p is reduced in *camta5&6* but over accumulated in *camta1* mutant. Thus, these results indicate that CAMTAs are, indeed, involved in the regulation of miRNA expression. The promoter of miR167h has one CAMTA-binding site, while that of miR156a harbors two sites. Furthermore, CAMTA4 appears to have a broader and major role, as it appears to regulate the expression of all the five miRNAs. In the case of miR156a, miR160a-5p, miR167h-5p, and miR168a-5p, it has a positive influence, while, for miR166a-3p, it acts to negatively regulate it. Taking this cue further, we ventured to find if the orthologs of these AtCAMTA4 and 6, i.e., OsCAMTA4 (LOC_Os04g31900) and OsCAMTA6 (LOC_Os07g43030) are involved in transcriptional regulation of miR156a and miR167h.

To find out whether OsCAMTA4 binds to the promoter of miR156a and miR167h, Y1H was performed using their promoter regions as bait. The promoter fragment containing the CAMTA-binding sites was cloned into the pABAi and transformed into Y1HGold to generate reporter strains. The basal expression of the bait reporter strain in the absence of prey was checked and found to be null. On the other hand, the full-length-coding sequence of OsCAMTA4 and 6 was cloned into pGAD7 to generate a prey vector. Both the prey vector and empty pGAD7 (as negative control) were transformed into Y1H gold strain-harboring promoter segments of miR156a and miR167h in separate experiments. Diploid yeast colonies containing miR156a promoter as bait and OsCAMTA4 and 6 as prey were able to grow strongly on the selective auxotrophic media-containing aureobasidin A. But miR167h shows a strong interaction with OsCAMTA6 and not with OsCAMTA4 (Figure 6B). Thus, hereby, the physical interaction of the CAMTA-binding sites residing in the promoters of miRNA156a, miR167h, with OsCAMTAs, was confirmed in rice.

**Figure 7:**
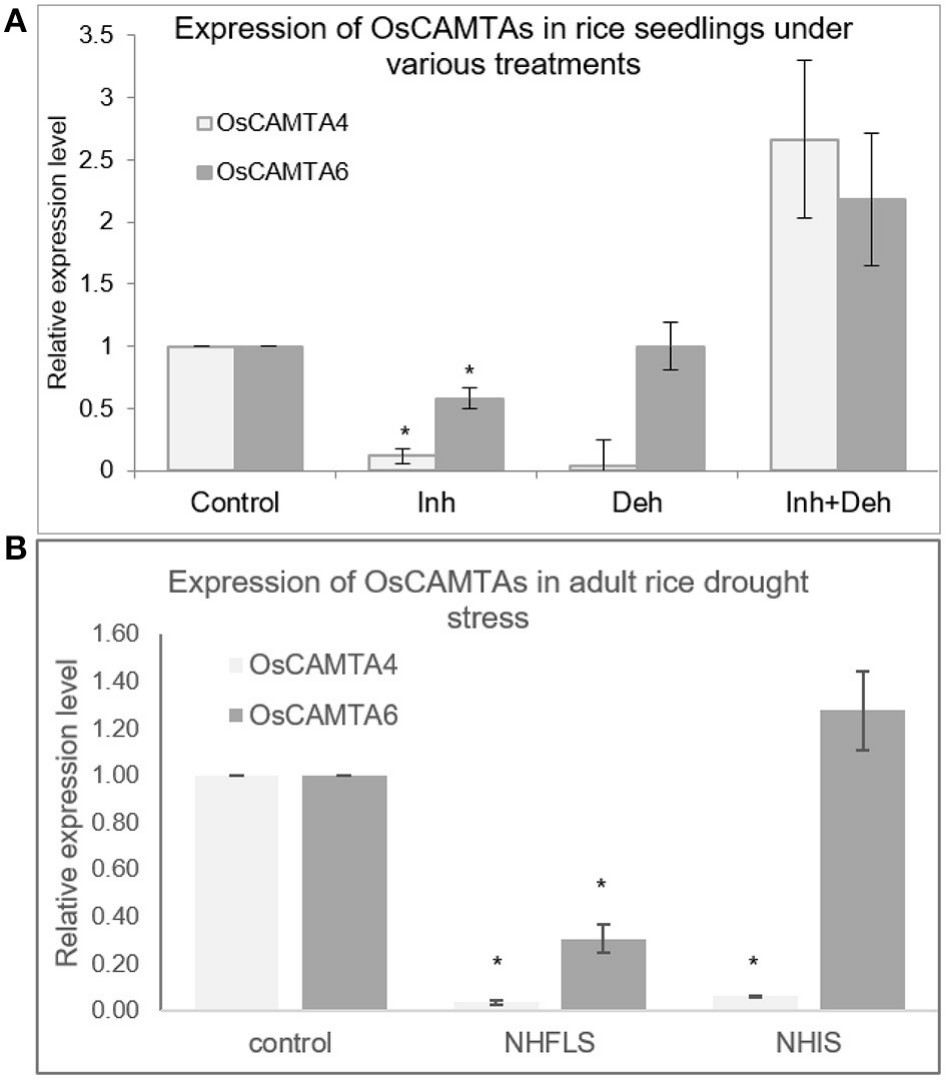
The expression profile of OsCAMTAs at different stages in rice. **(A)** The expression profile of *OsCAMTA4* and *OsCAMTA6* under conditions of control, [Ca^2+^]_cyt_ channel inhibition, dehydration, and dehydration preceded by [Ca^2+^]_cyt_ channel inhibition in N22 rice seedlings. **(B)** The expression profile of *OsCAMTA4* and *OsCAMTA6* under drought stress at the heading stage as measured in the flag leaf and inflorescence of N22 rice. The asterisks denote significant change as observed by the *t*-test.

Furthermore, expression patterns of OsCAMTA4 and 6 were checked in different tissues under control and stress conditions. Both the CAMTAs respond to inhibitor treatment in seedlings by the reduction in expression; however, their dehydration response could not be confirmed (Figure 7A). In the mature drought-stressed rice plant, both the miRNAs, miR156a, and miR167h-5p are downregulated greatly in Flag Leaf while remaining close to control in a spikelet (Balyan et al., 2017). Under the same drought conditions, both the CAMTAs are downregulated significantly in the Flag Leaf with only OsCAMTA4 reducing in inflorescence (Figure 7B). Notably, these two miRNAs (miR156a and miR167h-5p) appear to co-regulate with OsCAMTA4 during seedling dehydration stress as well as FL drought stress. Thus, OsCAMTA4 appears to be a major regulator of these miRNAs under different growth and environmental stimuli.

## Discussion

These regulatory small molecules named miRNAs are involved in almost all the critical biological processes in plants, including growth and development, as well as combating various stresses. While a significant number of studies have been done to understand the molecular and biochemical processes that are regulated by miRNAs, few studies attempt to study the regulation of miRNA genes themselves. To address this area, we investigated the molecular nature of the schema that regulates miRNA genes by looking into the involvement of one of the most important signaling molecules, i.e., cytosolic levels of calcium in regulating miRNA gene expression. In the past, Kaplan et al., 2006 studied the effect of [Ca^2+^]_cyt_ bursts induced by calmodulin inhibitors on gene expression of protein-coding genes in *Arabidopsis*. Over 200 genes were found to be differentially regulated (162 upregulated and 68 downregulated significantly), and it was summarized that [Ca^2+^]_cyt_ bursts in the cytosol affect the transcriptome to a certain extent and are responsible for the induction of signaling-related genes. In another study, where different types of [Ca^2+^]_cyt_ bursts were given (single peak, oscillations, and prolonged [Ca^2+^]_cyt_ elevation) by electrical stimulus, changes in gene expression were observed in *Arabidopsis* (Whalley et al., 2011). Herein again, the authors found that more genes were upregulated than downregulated in response to [Ca^2+^]_cyt_ single peak burst (104 up vs. 30 downregulated genes) and oscillations (256 upregulated vs. 97 downregulated genes) with a large number of overlapping genes among the upregulated ones. In our study as well, we found that miRNAs, indeed, respond to [Ca^2+^]_cyt_ levels. The proportion of such miRNAs in N22 (~2.8%) is pretty close to the estimate of protein-coding genes found by Kaplan et al. in *Arabidopsis* (~3%) and by Feske et al. (2001) in human T lymphocyte cells (~2.1%). Additionally, a higher proportion of miRNAs was upregulated by [Ca^2+^]_cyt_ than downregulated, which is similar to the findings mentioned above. Among the [Ca^2+^]_cyt_-responsive miRNAs as well, a big proportion of miRNAs (such as miR156, miR159, miR166, miR167, miR319, and miR396) is also responsive to, at least, one or multiple abiotic stresses, such as cold, drought, salt, UV, and mechanical (Balyan et al., 2015).

The data described above identified several miRNAs whose expression is regulated by cytosolic calcium levels. On the other hand, it is also possible that miRNAs target genes involved in the calcium-signaling cascade. Although prediction-based models have specified [Ca^2+^]_cyt_-signaling components as miRNA targets for a long time now (Sunkar et al., 2008), our study identifies those targets on the basis of degradome data. Data analysis identified genes such as IQ CaM-binding motif family protein (miR531b), OsCML16 (miR5517), OsCML22 (miR164a, c, d, and e), CAMK_CAMK_like.9 (miR2090), CAMK_CAMK_like.11 (miR5151), and MAPKKK (miR1428e-5p, miR2876-3p). In an earlier study in tomatoes, an autoinhibited [Ca^2+^]_cyt_ ATPase *ACA10* has been shown to be targeted by miR4376. This miR-target module is involved in proper fruit development since the overexpression of either the miRNA or the miR-resistant ACA10 resulted in elongated stamen filaments and a reduced number of mature fruits (Wang et al., 2011). Thus, it appears that miRNAs play an important role in the calcium-mediated regulatory schema of the plant cell.

[Ca^2+^]_cyt_ is known to be the mediator of several biotic and abiotic stress responses like heat, drought, touch, cold, and salinity (Knight et al., 1996; Albrecht et al., 2003; Choi et al., 2005; Wu et al., 2012; Schulz et al., 2013). In this study, we show that [Ca^2+^]_cyt_ also mediates the dehydration response of several miRNA genes as well. These included miRNAs like miR156a, miR167h-5p, miR168a-5p, and miR5788. Among these, the former two are also responsive to resting [Ca^2+^]_cyt_ under non-stress conditions, while the latter two are not. The trend exhibited by the latter group could be speculated by the involvement of some other factors such as dehydration-specific transcription factors that might be controlled by [Ca^2+^]_cyt_ signaling. Earlier, H_2_S, which is a potent-signaling messenger in various plant physiological processes, including drought (Jin et al., 2011, 2013), has been shown to mediate drought response of some miRNAs like miR167a/c/d, miR393a, miR396a, and miR398a/b/c in *Arabidopsis* (Shen et al., 2013). Another significant hormone known to be involved in many abiotic stresses, especially drought/dehydration stress—the abscisic acid and its influence on miRNA expression—has also been discussed in this study. In general, [Ca^2+^]_cyt_ is known to mediate many ABA responses, such a stomatal closure and seedling growth (McAinsh et al., 1990; Guo et al., 2002), and, thus, there is a significant overlap of ABA and calcium-mediated signaling. Indeed, the expression of several miRNAs was found to be regulated by ABA. Expression trends of miR1425-5p, miR156a, miR162b, miR168a, miR319b, miR528-5p, and miR530-5p match with previously known knowledge, while the rest had no previous information on their ABA response (Shen et al., 2010; Tian et al., 2015). Further analysis conducted to decipher whether modulation in the cytosolic calcium levels mediates ABA inducibility of the miRNAs resulted in the finding that cytosolic calcium levels do, indeed, mediate the ABA response of miR159b, miR319b, and miR530-5p.

Further investigation into the signaling cascade regulating the miRNA expression revealed the involvement of calmodulins (miR156a, miR1878, miR396c-3p, miR166g-3p, miR167h-5p, miR1425-5p, and miR1876). Calmodulins are signal sensor-relay proteins that directly bind to calcium and relay the signal to other proteins, such as CAMTAs (calmodulin-binding transcription activators), that bind to particular motifs in promoter sequences of genes and either activate or suppress transcription. The use of calmodulin inhibitors and *camta* knockdown mutants affected the expression of certain miRNAs, namely, miR156a, miR160a-5p, miR166a-3p, miR167h-5p, and miR168a-5p. The effect of knockdown of *camta*s on the miRNA expression in the mutants can be a result of direct interaction between the TF and the miRNA promoter or it can be routed *via* some other TFs/proteins that might be regulated by the CAMTAs. CAMTAs have been previously shown to mediate cold response as well as salicylic acid-mediated immunity in *Arabidopsis*. However, their involvement in miRNA regulation is not well-known in plants. Besides, several calcium-responsive promoter motifs have been identified in the promoter sequences of calcium-responsive genes (Finkler et al., 2006; Kaplan et al., 2006; Galon et al., 2010). Accordingly, we found the presence of these calcium-responsive motifs, namely, CARE (calcium-responsive elements), ABRE (ABA-responsive elements and related motifs), E-box, G-box, GT-box, Z-box, and CAMTA-binding sites in the promoter sequences of calcium-responsive miRNAs. The Y1H shows strong physical interaction between OsCAMTA4 and miR156a, but this CAMTA does not interact with miR167h. However, OsCAMTA6 shows strong interaction with the promoters of miR156a and miR167h. Thus, hereby, we reveal novel and key players in the transcriptional regulation of miR156a and miR167h. While miR156a is seen to be responsive to [Ca^2+^]_cyt_ in resting conditions, positively by calmodulin as well as [Ca^2+^]-mediated dehydration, miR167h-5p has a slightly different story. Although [Ca^2+^]_cyt_ affects its expression under control conditions as well as dehydration, miR167h-5p shows negative regulation by calmodulins. Both of these miRNAs show binding and trans-activation by OsCAMTA4, which appears to be co-regulated in seedling dehydration as well as FL drought stress. This appears as a significant finding since miRNA expression has to date not been associated with CAMTAs.

miR156 has been studied in quite a detail, and there is plenty of knowledge available about its function and regulation. The miRNA is a floral repressor, and promoter of the juvenile phase in *Arabidopsis* (Wu G. et al., 2009) is involved in lateral root development and leaf morphology in *Arabidopsis* (Gao et al., 2018). Regarding its regulation, it has been shown that AGL15 and AGL18 act in cooperation to promote its transcription by binding to the CArG motifs in its promoter (Serivichyaswat et al., 2015). The phytochrome-interacting factors or PIF1, PIF3, PIF4, and PIF5 have also been shown to directly bind and repress the expression of miR156b/d/e/f/h to enhance the shade avoidance syndrome in *Arabidopsis*. Another protein called DOG1 (DELAY OF GERMINATION 1) plays a role in the efficient processing of primary miR156 to its active mature form by regulating the processing proteins—DCL1, HYL1, SE, TGH, and CDC5 (Huo et al., 2016). Recently, another player has been found in miR159 that targets MYB33 that, in turn, binds and promotes the transcription of miR156a&c during the young seedling stage (Guo et al., 2017). Furthermore, clues behind its temporal expression pattern have been found in epigenetic regulation. The transcription-activating mark H3K4me3 is seen abundantly at the miR156a and miR156c loci during the early seedling stages wherein it contributes to its high expression (Xu et al., 2018), while, during the vegetative phase change, there is an increase in the level of histone H3K27me3 with a simultaneous decrease in H3K4me4 and H3K27ac at regions upstream and immediately downstream of its TSS, resulting in its decline in abundance (Xu et al., 2016). Besides, a cycling DOF transcription factor CDF2 has also been shown to be its transcriptional activator (and repressor of miR172) acting in the same signaling pathway to control flowering in *Arabidopsis* (Sun et al., 2015). Regarding the regulation of miR156 in rice, drought has already been shown to downregulate the miRNA in inflorescence tissue (Zhou L. et al., 2010). Besides, it is also responsive to several hormones, such as auxin in *Arabidopsis* [downregulation; (Marin et al., 2010)] and ethylene in tomato [downregulation; (Zuo et al., 2012)]. In rice, the miRNA has been shown to be independent of any regulation by gibberellin during juvenile to the adult phase transition (Tanaka, 2012). Our data bring more regulators of miR156 into light, which are [Ca^2+^] and OsCAMTA4 and 6. Since these are located pretty high in the signaling hierarchy that generates any responses inside a cell, this new mode of regulation might help to explain the various responses miR156 displays under the various abiotic stresses and different stages of the lifecycle of the plant.

miR167 is also a conserved miRNA across monocots and dicots and is known to target the ARF6/8 genes, thereby acting as a node in auxin signaling (Barik et al., 2015). It is known to be involved in adventitious root development in rice (Meng et al., 2011), modulation of auxin signaling during bacterial infection in tomato (Jodder et al., 2017), in regenerating calli in rice (Sinha et al., 2019), in blue-light signaling in *Arabidopsis* (Pashkovskiy et al., 2016), and abiotic stress, such as salinity (Liu et al., 2008; Ding et al., 2009; Frazier et al., 2011) and drought in rice (Balyan et al., 2017). For such an evolutionarily conserved and functionally significant miRNA, the information about its regulation was lacking. In our study, we showed its regulation *via* [Ca^2+^]_cyt_, calmodulin, and OsCAMTA6. Its dehydration response is also mediated *via* ${\text{[Ca]}}_{\text{cyt}}^{2+}$. Thus, it is a remarkable discovery for a miRNA that acts as a node in a critical signaling pathway such as the auxin signaling, ultimately regulating a plethora of plant functions.

## Conclusions

The understanding of the regulatory pathways governing the expression of another class of regulators, i.e., the miRNAs is critical in order to be able to manipulate them for advantageous traits in plants. With this view, the study explored the involvement of cytosolic calciumin-regulating expression of miRNA genes under control and drought stress conditions that we could establish with the help of calcium channel inhibitors and ionophores. The fact that ${\text{[Ca]}}_{\text{cyt}}^{2+}$ mediates several abiotic responses was also explored and it was demonstrated, it is the same for miRNAs in rice as well. The further dissection revealed the involvement of calmodulins and CAMTAs. Through yeast-one-hybrid experiments, OsCAMTA4 and 6 were proved to bind the CAMTA-binding sites of the very critical miR156a and miR167h. Both these CAMTAs were found to be coregulated with these two miRNAs at various developmental and stress stages indicative of a possible regulatory schema for these miRNAs in rice.

## Data Availability Statement

The data presented in the study are deposited in NCBI under the accession number PRJEB47136.

## Author Contributions

SR conceived the concept. SR and SK designed the experiments and prepared the manuscript. SK constructed the NGS libraries and performed the data analysis and qRT-PCR. VP performed qRT-PCR and yeast one-hybrid experiments. RM performed the degradome analysis. All the authors read and approved the final manuscript.

## Funding

The study was funded by Science and Engineering Research Board, Department of Science and Technology under the Grant No: EMR/2016/006081 awarded to the corresponding author.

## Conflict of Interest

The authors declare that the research was conducted in the absence of any commercial or financial relationships that could be construed as a potential conflict of interest.

## Publisher's Note

All claims expressed in this article are solely those of the authors and do not necessarily represent those of their affiliated organizations, or those of the publisher, the editors and the reviewers. Any product that may be evaluated in this article, or claim that may be made by its manufacturer, is not guaranteed or endorsed by the publisher.

## Supplementary Material

Supplementary Figure 1 A line diagram depicting the promoter sites cloned for the Y-1H experiment.

Supplementary Figure 2 Expression of OsCPK6 in response to various treatments under study. OsCPK6 has been considered as a marker for an efficiency check of calcium scarcity and excess environments created by the use of calcium channel inhibitors and ionophores.

Supplementary Figure 3 The expression profile of the three main miRNA-processing enzymes, namely *DCL1, HYL1*, and *SERRATE* under Ca^2+^ scarcity generated by the use of channel inhibitors or excess generated by treatment with calcium ionophore.

Supplementary Figure 4 Expression of *CAMTA* genes in their respective mutants.

Supplementary Table 1 A list of primers used in the study.

Supplementary Table 2 Details of the small RNA libraries generated for the various treatments given to 1-week-old N22 rice seedlings.

Supplementary Table 3 NGS data of all the miRNAs detected in response to calcium channel inhibitors in N22.

Supplementary Table 4a NGS data of all the miRNAs detected in response to dehydration in N22.

Supplementary Table 4b NGS data of all the miRNAs detected in response to inhibitor+dehydration in N22.

## Abbreviations

ABA: Abscisic acid
[Ca^2+^]_cyt_: cytosolic calcium
CAMTA: Calmodulin-binding transcription activators
CaM: Calmodulin
sRNA: small RNA.

## References

[B1] Albrecht V. Weinl S. Blazevic D. D'Angelo C. Batistic O. Kolukisaoglu Ü.. (2003). The calcium sensor CBL1 integrates plant responses to abiotic stresses. Plant J. 36, 457–470. 10.1046/j.1365-313X.2003.01892.x 14617077

[B2] Balyan S. Kumar M. Mutum R. D. Raghuvanshi U. Agarwal P. Mathur S.. (2017). Identification of miRNA-mediated drought responsive multi-tiered regulatory network in drought tolerant rice, Nagina. Sci. Rep. 7, 1–17. 10.1038/s41598-017-15450-1 29133823 PMC5684420

[B3] Balyan S. C. Mutum R. D. Kansal S. Kumar S. Raghuvanshi S. (2015). Insights into the small RNA-mediated networks in response to abiotic stress in plants, in Elucidation of Abiotic Stress Signaling in Plants: Functional Genomics Gerspectives, Vol. 2, ed. G. K. Pandey (New York, NY: Springer), 45–92. 10.1007/978-1-4939-2540-7_3

[B4] Barik S. Kumar A. Sarkar Das S. Yadav S. Gautam V. Singh A.. (2015). Coevolution pattern and functional conservation or divergence of miR167s and their targets across Diverse Plant Species. Sci. Rep. 5:14611. 10.1038/srep14611 26459056 PMC4602222

[B5] Chen H. Yang Q. Chen K. Zhao S. Zhang C. Pan R.. (2019). Integrated microRNA and transcriptome profiling reveals a miRNA-mediated regulatory network of embryo abortion under calcium deficiency in peanut (*Arachis hypogaea* L.). *BMC Genomics* 20:392. 10.1186/s12864-019-5770-6 31113378 PMC6528327

[B6] Choi M. S. Kim M. C. Yoo J. H. Moon B. C. Koo S. C. Park B. O.. (2005). Isolation of a calmodulin-binding transcription factor from rice (*Oryza* sativa L.). J. Biol. Chem. 280, 40820–40831. 10.1074/jbc.M504616200 16192280

[B7] Ding D. Zhang L. Wang H. Liu Z. Zhang Z. Zheng Y. (2009). Differential expression of miRNAs in response to salt stress in maize roots. Ann. Bot. 103, 29–38. 10.1093/aob/mcn205 18952624 PMC2707283

[B8] Dodd A. N. Kudla J. Sanders D. (2010). The language of calcium signaling. Annu. Rev. Plant Biol. 61, 593–620. 10.1146/annurev-arplant-070109-104628 20192754

[B9] Doherty C. J. Van Buskirk H. A. Myers S. J. Thomashow M. F. (2009). Roles for Arabidopsis CAMTA transcription factors in cold-regulated gene expression and freezing tolerance. Plant Cell 21, 972–984. 10.1105/tpc.108.063958 19270186 PMC2671710

[B10] Du L. Ali G. S. Simons K. A. Hou J. Yang T. Reddy A. S. N.. (2009). Ca2+/calmodulin regulates salicylic-acid-mediated plant immunity. Nature 457, 1154–1158. 10.1038/nature07612 19122675

[B11] Feske S. Giltnane J. Dolmetsch R. Staudt L. M. Rao A. (2001). Gene regulation mediated by calcium signals in T lymphocytes. Nat. Immunol. 2, 316–324. 10.1038/86318 11276202

[B12] Finkler A. Kaplan B. Fromm H. (2006). Ca2+ - responsive cis-elements in palnts. Plant Cell 18, 2733–2748. 16980540 10.1105/tpc.106.042713 PMC1626612

[B13] Franklin-Tong V. E. Ride J. P. Read N. D. Trewavas A. J. Christopher F. (1993). The self-incompatibility response in Papaver rhoeas is mediated by cytosolic free calcium. Plant J. 4, 163–177. 10.1046/j.1365-313X.1993.04010163.x

[B14] Frazier T. P. Sun G. Burklew C. E. Zhang B. (2011). Salt and drought stresses induce the aberrant expression of microRNA genes in tobacco. Mol. Biotechnol. 49, 159–165. 10.1007/s12033-011-9387-5 21359858

[B15] Galon Y. Finkler A. Fromm H. (2010). Calcium-regulated transcription in plants. Mol. Plant 3, 653–669. 10.1093/mp/ssq019 20457642

[B16] Gao R. Wang Y. Gruber M. Y. Hannoufa A. (2018). MiR156/SPL10 modulates lateral root development, branching and leaf morphology in arabidopsis by silencing AGAMOUS-LIKE 79. Front. Plant Sci. 8:2226. 10.3389/fpls.2017.02226 29354153 PMC5758603

[B17] Gao Y. Zhang G. (2019). A calcium sensor calcineurin B-like 9 negatively regulates cold tolerance via calcium signaling in *Arabidopsis thaliana*. Plant Signal. Behav. 14, 1–6. 10.1080/15592324.2019.1573099 30696338 PMC6422375

[B18] Gaupels F. Kuruthukulangarakoola G. T. Durner J. (2011). Upstream and downstream signals of nitric oxide in pathogen defence. Curr. Opin. Plant Biol. 14, 707–714. 10.1016/j.pbi.2011.07.005 21816662

[B19] Guo C. Xu Y. Shi M. Lai Y. Wu X. Wang H.. (2017). Repression of miR156 by miR159 regulates the timing of the juvenile-to-adult transition in arabidopsis. Plant Cell 29, 1293–1304. 10.1105/tpc.16.00975 28536099 PMC5502449

[B20] Guo Y. Xiong L. Song C. P. Gong D. Halfter U. Zhu J. K. (2002). A calcium sensor and its interacting protein kinase are global regulators of abscisic acid signaling in Arabidopsis. Dev. Cell 3, 233–244. 10.1016/S1534-5807(02)00229-0 12194854

[B21] Hepler P. K. Vidali L. Cheung A. Y. (2001). Polarized cell growth in higher plants. Annu. Rev. Cell Dev. Biol. 17, 159–187. 10.1146/annurev.cellbio.17.1.159 11687487

[B22] Hsieh H. L. Song C. J. Roux S. J. (2000). Regulation of a recombinant pea nuclear apyrase by calmodulin and casein kinase II. Biochim. Biophys. Acta - Gene Struct. Expr. 1494, 248–255. 10.1016/S0167-4781(00)00245-1 11121582

[B23] Hu X. Y. Neill S. J. Cai W. M. Tang Z. C. (2004). Induction of defence gene expression by oligogalacturonic acid requires increases in both cytosolic calcium and hydrogen peroxide in Arabidopsis thaliana. Cell Res. 14, 234–240. 10.1038/sj.cr.7290224 15225417

[B24] Huo H. Wei S. Bradford K. J. (2016). DELAY of GERMINATION1 (DOG1) regulates both Seed dormancy and flowering time through microRNA pathways. Proc. Natl. Acad. Sci. U. S. A. 113, E2199–E2206. 10.1073/pnas.1600558113 27035986 PMC4839450

[B25] Jin Z. Shen J. Qiao Z. Yang G. Wang R. Pei Y. (2011). Hydrogen sulfide improves drought resistance in Arabidopsis thaliana. Biochem. Biophys. Res. Commun. 414, 481–486. 10.1016/j.bbrc.2011.09.090 21986537

[B26] Jin Z. Xue S. Luo Y. Tian B. Fang H. Li H.. (2013). Hydrogen sulfide interacting with abscisic acid in stomatal regulation responses to drought stress in Arabidopsis. Plant Physiol. Biochem. 62, 41–46. 10.1016/j.plaphy.2012.10.017 23178483

[B27] Jodder J. Basak S. Das R. Kundu P. (2017). Coherent regulation of miR167a biogenesis and expression of auxin signaling pathway genes during bacterial stress in tomato. Physiol. Mol. Plant Pathol. 100, 97–105. 10.1016/j.pmpp.2017.08.001

[B28] Kansal S. Devi R. M. Balyan S. C. Arora M. K. Singh A. K. Mathur S.. (2015). Unique miRNome during anthesis in drought-tolerant indica rice var. Nagina 22. Planta 24, 1543–1559. 10.1007/s00425-015-2279-3 25809150

[B29] Kaplan B. Davydov O. Knight H. Galon Y. Knight M. R. Fluhr R.. (2006). Rapid transcriptome changes induced by cytosolic Ca2+ transients reveal ABRE-related sequences as Ca2+-responsive cis elements in Arabidopsis. Plant Cell 18, 2733–2748. 10.1105/tpc.106.042713 16980540 PMC1626612

[B30] Kim Y. S. An C. Park S. Gilmour S. J. Wang L. Renna L.. (2017). CAMTA-mediated regulation of salicylic acid immunity pathway genes in arabidopsis exposed to low temperature and pathogen infection. Plant Cell 29, 2465–2477. 10.1105/tpc.16.00865 28982964 PMC5774559

[B31] Knight H. Trewavas A. J. Knight M. R. (1996). Cold calcium signaling in arabidopsis lnvolves two cellular pools and a change in calcium signature after Acclimation. Plant Cell 8, 489–503. 10.1105/tpc.8.3.489 8721751 PMC161115

[B32] Kushwaha R. Singh A. Chattopadhyay S. (2008). Calmodulin7 plays an important role as transcriptional regulator in arabidopsis seedling development. Plant Cell 20, 1747–1759. 10.1105/tpc.107.057612 18621945 PMC2518239

[B33] Legué V. Biancaflor E. Wymer C. Perbal G. Fantin D. Gilroy S. (1997). Cytoplasmic free Ca2+ in arabidopsis roots changes in response to touch but not gravity. Plant Physiol. 114, 789–800. 10.1104/pp.114.3.789 9232870 PMC158365

[B34] Li T. Li H. Zhang Y. X. Liu J. Y. (2011). Identification and analysis of seven H2O2-responsive miRNAs and 32 new miRNAs in the seedlings of rice (*Oryza sativa* L. ssp. indica). Nucleic Acids Res. 39, 2821–2833. 10.1093/nar/gkq1047 21113019 PMC3074118

[B35] Li Y. F. Zheng Y. Addo-Quaye C. Zhang L. Saini A. Jagadeeswaran G.. (2010). Transcriptome-wide identification of microRNA targets in rice. Plant J. 62, 742–759. 10.1111/j.1365-313X.2010.04187.x 20202174

[B36] Lisjak M. Teklic T. Wilson I. D. Whiteman M. Hancock J. T. (2013). Hydrogen sulfide: environmental factor or signalling molecule? Plant, Cell Environ. 36, 1607–1616. 10.1111/pce.12073 23347018

[B37] Liu H. H. Tian X. Li Y. J. Wu C. A. Zheng C. C. (2008). Microarray-based analysis of stress-regulated microRNAs in *Arabidopsis thaliana*. RNA 14, 836–843. 10.1261/rna.895308 18356539 PMC2327369

[B38] Ma W. Berkowitz G. A. (2007). The grateful dead: calcium and cell death in plant innate immunity. Cell. Microbiol. 9, 2571–2585. 10.1111/j.1462-5822.2007.01031.x 17714518

[B39] Marin E. Jouannet V. Herz A. Lokerse A. S. Weijers D. Vaucheret H.. (2010). mir390, Arabidopsis TAS3 tasiRNAs, and their AUXIN RESPONSE FACTOR targets define an autoregulatory network quantitatively regulating lateral root growth. Plant Cell 22, 1104–1117. 10.1105/tpc.109.072553 20363771 PMC2879756

[B40] McAinsh M. R. Brownlee C. Hetherington A. M. (1990). Abscisic acid-induced elevation of guard cell cytosolic Ca2+ precedes stomatal closure. Nature 343, 186–188. 10.1038/343186a0

[B41] Meng Y. Shao C. Wang H. Chen M. (2011). The regulatory activities of plant microRNAs: a more dynamic perspective. Plant Physiol. 157, 1583–1595. 10.1104/pp.111.187088 22003084 PMC3327222

[B42] Mittler R. Vanderauwera S. Suzuki N. Miller G. Tognetti V. B. Vandepoele K.. (2011). ROS signaling: the new wave? Trends Plant Sci. 16, 300–309. 10.1016/j.tplants.2011.03.007 21482172

[B43] Mutum R. D. Balyan S. C. Kansal S. Agarwal P. Kumar S. Kumar M.. (2013). Evolution of variety-specific regulatory schema for expression of osa-miR408 in indica rice varieties under drought stress. FEBS J. 280, 1717–1730. 10.1111/febs.12186 23399101

[B44] Mutum R. D. Kumar S. Balyan S. Kansal S. Mathur S. Raghuvanshi S. (2016). Identification of novel miRNAs from drought tolerant rice variety Nagina 22. Sci. Rep. 6:30786. 10.1038/srep30786 27499088 PMC4976344

[B45] Park C. Y. Lee J. H. Yoo J. H. Moon B. C. Choi M. S. Kang Y. H.. (2005). WRKY group IId transcription factors interact with calmodulin. FEBS Lett. 579, 1545–1550. 10.1016/j.febslet.2005.01.057 15733871

[B46] Pashkovskiy P. P. Kartashov A. V. Zlobin I. E. Pogosyan S. I. Kuznetsov V. V. (2016). Blue light alters miR167 expression and microRNA-targeted auxin response factor genes in *Arabidopsis thaliana* plants. Plant Physiol. Biochem. 104, 146–154. 10.1016/j.plaphy.2016.03.018 27031426

[B47] Pauly N. Knight M. R. Thuleau P. Graziana A. Muto S. Ranjeva R.. (2001). The nucleus together with the cytosol generates patterns of specific cellular calcium signatures in tobacco suspension culture cells. Cell Calcium 30, 413–421. 10.1054/ceca.2001.0250 11728136

[B48] Pauly N. Knight M. R. Thuleau P. Van Der Luit A. H. Moreau M. Trewavas A. J.. (2000). Control of free calcium in plant cell nuclei. Nature 405, 754–755. 10.1038/35015671 10866186

[B49] Reddy A. S. N. Day I. S. Narasimhulu S. B. Safadi F. Reddy V. S. Golovkin M.. (2002). Isolation and characterization of a novel calmodulin-binding protein from potato. J. Biol. Chem. 277, 4206–4214. 10.1074/jbc.M104595200 11684678

[B50] Schulz P. Herde M. Romeis T. (2013). Calcium-dependent protein kinases: hubs in plant stress signaling and development. Plant Physiol. 163, 523–530. 10.1104/pp.113.222539 24014579 PMC3793034

[B51] Sedbrook J. C. Kronebusch P. J. Borisy G. G. Trewavas A. J. Masson P. H. (1996). Transgenic AEQUORIN reveals organ-specific cytosolic Ca2+ responses to anoxia in *Arabidopsis thaliana* seedling. Plant Physiol. 111, 243–257. 10.1104/pp.111.1.243 8685265 PMC157832

[B52] Serivichyaswat P. Ryu H. S. Kim W. Kim S. Chung K. S. Kim J. J.. (2015). Expression of the floral repressor miRNA156 is positively regulated by the AGAMOUS-like proteins AGL15 and AGL18. Mol. Cells 38, 259–266. 10.14348/molcells.2015.2311 25666346 PMC4363726

[B53] Shen J. Xie K. Xiong L. (2010). Global expression profiling of rice microRNAs by one-tube stem-loop reverse transcription quantitative PCR revealed important roles of microRNAs in abiotic stress responses. Mol. Genet. Genomics 284, 477–488. 10.1007/s00438-010-0581-0 20941508

[B54] Shen J. Xing T. Yuan H. Liu Z. Jin Z. Zhang L.. (2013). Hydrogen Sulfide Improves Drought Tolerance in *Arabidopsis thaliana* by MicroRNA Expressions. PLoS One 8, 4–11. 10.1371/journal.pone.0077047 24194857 PMC3806758

[B55] Sinha A. Solanki M. Shukla L. I. (2019). Evidences for differential expression of miR167d-5p, target, positional nucleotide preference, and its role in somatic and different stages of regenerating calli of *Oryza sativa*. Plant Cell. Tissue Organ Cult. 136, 537–548. 10.1007/s11240-018-01535-w

[B56] Song S. Jia Z. Xu J. Zhang Z. Bian Z. (2011). N-butyryl-homoserine lactone, a bacterial quorum-sensing signaling molecule, induces intracellular calcium elevation in Arabidopsis root cells. Biochem. Biophys. Res. Commun. 414, 355–360. 10.1016/j.bbrc.2011.09.076 21964296

[B57] Sun Z. Guo T. Liu Y. Liu Q. Fang Y. (2015). The roles of arabidopsis CDF2 in transcriptional and posttranscriptional regulation of primary microRNAs. PLoS Genet. 11:e1005700. 10.1371/journal.pgen.1005700 26473486 PMC4608766

[B58] Sunkar R. Zhou X. Zheng Y. Zhang W. Zhu J. K. (2008). Identification of novel and candidate miRNAs in rice by high throughput sequencing. BMC Plant Biol. 8:25. 10.1186/1471-2229-8-25 18312648 PMC2292181

[B59] Tanaka N. (2012). Gibberellin is not a regulator of miR156 in rice juvenile-adult phase change. Rice 5, 1–6. 10.1186/1939-8433-5-25 24279896 PMC4883733

[B60] Tian C. Zuo Z. Qiu J. L. (2015). Identification and characterization of ABA-responsive microRNAs in rice. J. Genet. Genomics 42, 393–402. 10.1016/j.jgg.2015.04.008 26233894

[B61] Wan B. Lin Y. Mou T. (2007). Expression of rice Ca2+-dependent protein kinases (CDPKs) genes under different environmental stresses. FEBS Lett. 581, 1179–1189. 10.1016/j.febslet.2007.02.030 17336300

[B62] Wang Y. Bai X. Yan C. Gui Y. Wei X. Zhu Q. H.. (2012). Genomic dissection of small RNAs in wild rice (*Oryza rufipogon*): lessons for rice domestication. New Phytol. 196, 914–925. 10.1111/j.1469-8137.2012.04304.x 22994911

[B63] Wang Y. Itaya A. Zhong X. Wu Y. Zhang J. van der Knaap E.. (2011). Function and evolution of a microRNA that regulates a caspi2+-ATPase and triggers the formation of phased small interfering rnas in tomato reproductive Growth. Plant Cell 23, 3185–3203. 10.1105/tpc.111.088013 21917547 PMC3203446

[B64] Whalley H. J. Sargeant A. W. Steele J. F. C. Lacoere T. Lamb R. Saunders N. J.. (2011). Transcriptomic analysis reveals calcium regulation of specific promoter motifs in arabidopsis. Plant Cell 23, 4079–4095. 10.1105/tpc.111.090480 22086087 PMC3246331

[B65] Wu B. Wang M. Ma Y. Yuan L. Lu S. (2012). High-throughput sequencing and characterization of the small RNA Transcriptome reveal features of novel and conserved microRNAs in *Panax ginseng*. PLoS One 7:e44385. 10.1371/journal.pone.0044385 22962612 PMC3433442

[B66] Wu G. Park M. Y. Conway S. R. Wang J.-W. Weigel D. Poethig R. S. (2009). The sequential action of miR156 and miR172 regulates developmental timing in Arabidopsis. Cell 6, 1249–1254. 10.1016/j.cell.2009.06.031 19703400 PMC2732587

[B67] Wu L. Zhang Q. Zhou H. Ni F. Wu X. Qi Y. (2009). Rice microrna effector complexes and targets. Plant Cell 21, 3421–3435. 10.1105/tpc.109.070938 19903869 PMC2798332

[B68] Xiong C. T. Jauneau A. Ranjeva R. Mazars C. (2004). Isolated plant nuclei as mechanical and thermal sensors involved in calcium signalling. Plant J. 40, 12–21. 10.1111/j.1365-313X.2004.02184.x 15361137

[B69] Xu M. Hu T. Zhao J. Park M. Y. Earley K. W. Wu G.. (2016). Developmental functions of miR156-regulated SQUAMOSA PROMOTER BINDING PROTEIN-LIKE (SPL) genes in *Arabidopsis thaliana*. PLoS Genet. 12:e1006263. 10.1371/journal.pgen.1006263 27541584 PMC4991793

[B70] Xu Y. Zhang L. Wu G. (2018). Epigenetic regulation of juvenile-to-adult transition in plants. Front. Plant Sci. 9:1048. 10.3389/fpls.2018.01048 30079076 PMC6063087

[B71] Yang J. Liu X. Xu B. Zhao N. Yang X. Zhang M. (2013). Identification of miRNAs and their targets using high-throughput sequencing and degradome analysis in cytoplasmic male-sterile and its maintainer fertile lines of brassica juncea. BMC Genomics 14:9. 10.1186/1471-2164-14-9 23324572 PMC3553062

[B72] Yang T. Poovaiah B. W. (2003). Calcium/calmodulin-mediated signal network in plants. Trends Plant Sci. 8, 505–512. 10.1016/j.tplants.2003.09.004 14557048

[B73] Zheng Y. Zhan Q. Shi T. Liu J. Zhao K. Gao Y. (2020). The nuclear transporter SAD2 plays a role in calcium- and H2O2-mediated cell death in Arabidopsis. Plant J. 101, 324–333. 10.1111/tpj.14544 31565820

[B74] Zhou L. Liu Y. Liu Z. Kong D. Duan M. Luo L. (2010). Genome-wide identification and analysis of drought-responsive microRNAs in Oryza sativa. J. Exp. Bot. 61, 4157–4168. 10.1093/jxb/erq237 20729483

[B75] Zhou M. Gu L. Li P. Song X. Wei L. Chen Z.. (2010). Degradome sequencing reveals endogenous small RNA targets in rice (*Oryza sativa* L. ssp. indica). Front. Biol. 5, 67–90. 10.1007/s11515-010-0007-8

[B76] Zuo J. Zhu B. Fu D. Zhu Y. Ma Y. Chi L.. (2012). Sculpting the maturation, softening and ethylene pathway: the influences of microRNAs on tomato fruits. BMC Genomics 13:7. 10.1186/1471-2164-13-7 22230737 PMC3266637

