# Supplementary material for: Investigations on Regulation of MicroRNAs in Rice Reveal [Ca2+]cyt Signal Transduction Regulated MicroRNAs
Source: Front Plant Sci. 2021 Oct 18;12:720009. doi: 10.3389/fpls.2021.720009 (PMC8558223; doi:10.3389/fpls.2021.720009)
Supplement: Supplementary Figure 1 — A line diagram depicting the promoter sites cloned for the Y-1H experiment. [file Presentation_1.PPTX]

## Slide 1
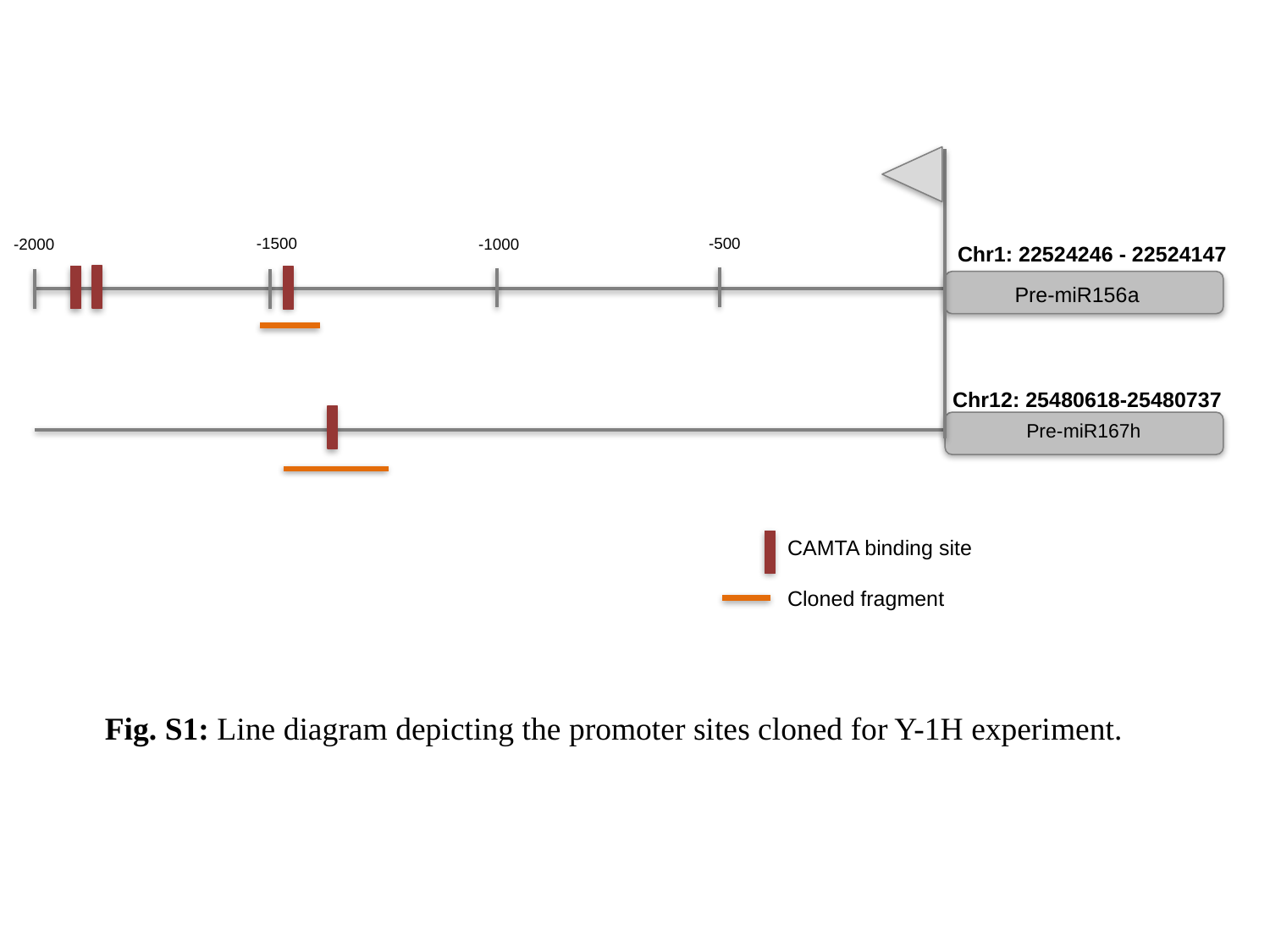

-1500
-500
-2000
-1000
Chr1: 22524246 - 22524147
Pre-miR156a
Chr12: 25480618-25480737
Pre-miR167h
CAMTA binding site
Cloned fragment
Fig. S1: Line diagram depicting the promoter sites cloned for Y-1H experiment.

## Slide 2
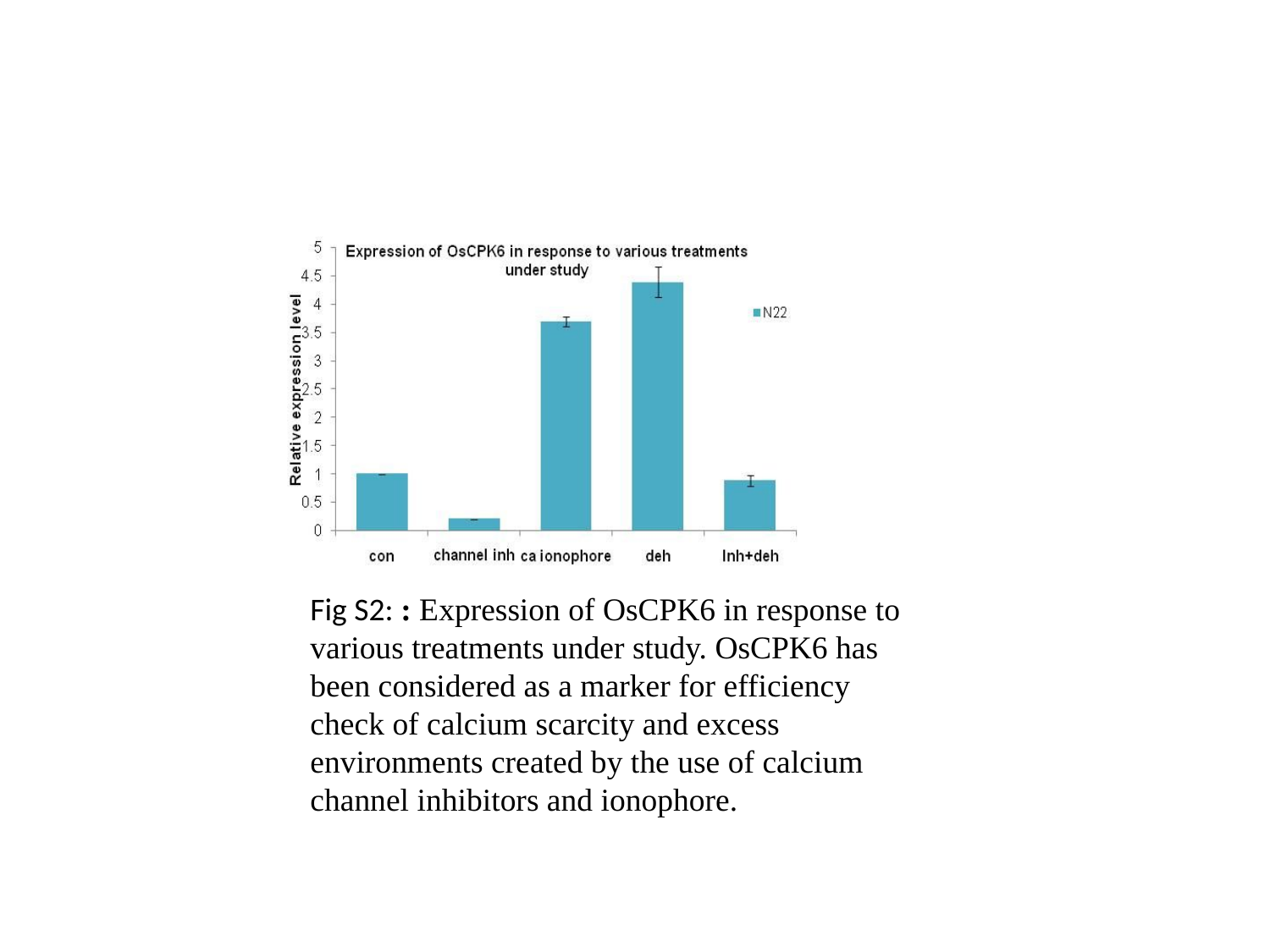

Fig S2: : Expression of OsCPK6 in response to various treatments under study. OsCPK6 has been considered as a marker for efficiency check of calcium scarcity and excess environments created by the use of calcium channel inhibitors and ionophore.

## Slide 3
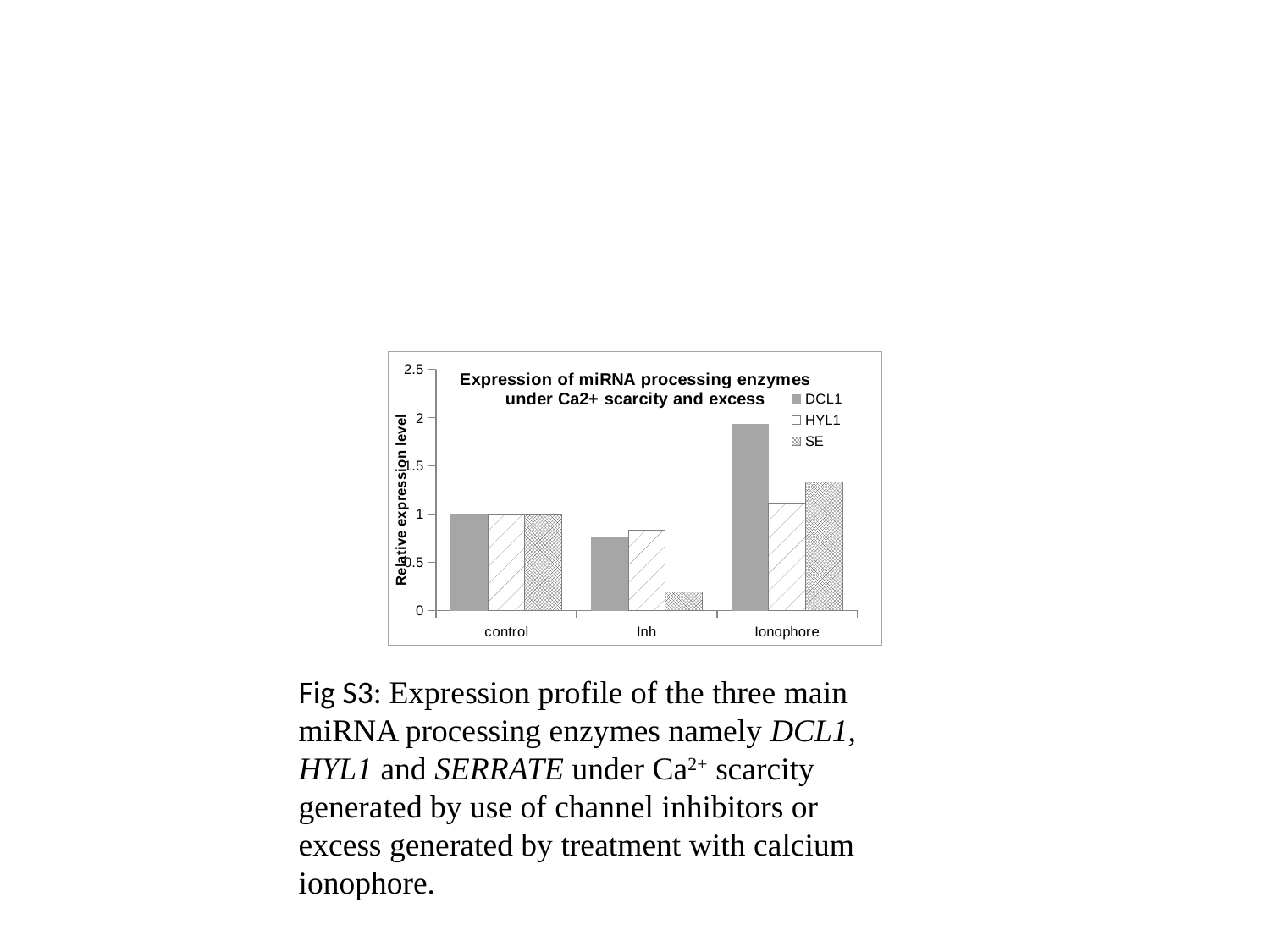

### Chart: Expression of miRNA processing enzymes under Ca2+ scarcity and excess
| Category | DCL1 | HYL1 | SE |
|---|---|---|---|
| control | 1.0 | 1.0 | 1.0 |
| Inh | 0.755701440993349 | 0.832253040533696 | 0.192062283737025 |
| Ionophore | 1.9328643 | 1.1133 | 1.3334 |Fig S3: Expression profile of the three main miRNA processing enzymes namely DCL1, HYL1 and SERRATE under Ca2+ scarcity generated by use of channel inhibitors or excess generated by treatment with calcium ionophore.

## Slide 4
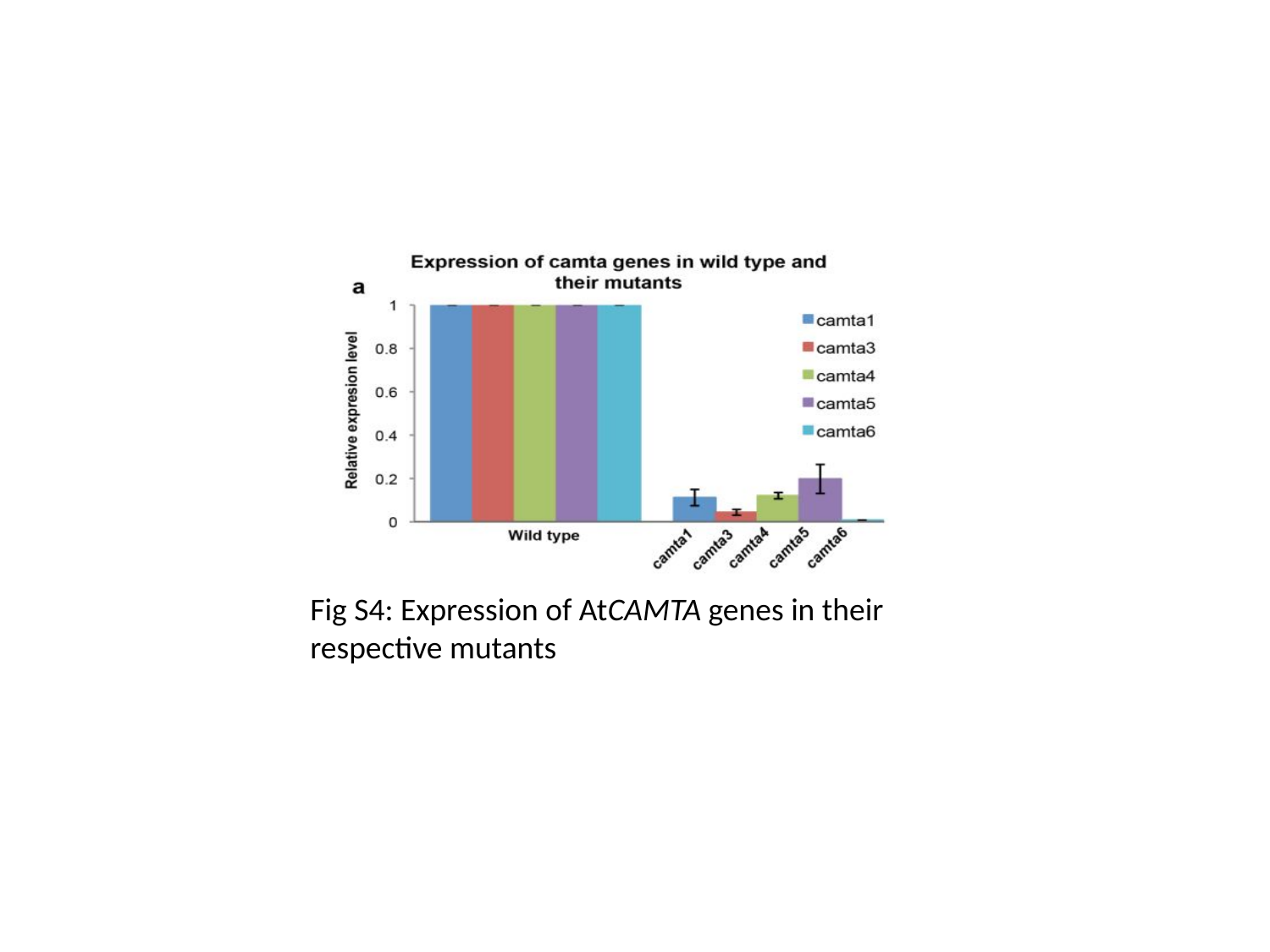

Fig S4: Expression of AtCAMTA genes in their respective mutants
